# Recombination rate variation shapes barriers to introgression across butterfly genomes

**DOI:** 10.1371/journal.pbio.2006288

**Published:** 2019-02-07

**Authors:** Simon H. Martin, John W. Davey, Camilo Salazar, Chris D. Jiggins

**Affiliations:** 1 Department of Zoology, University of Cambridge, Cambridge, United Kingdom; 2 Department of Biology, University of York, York, United Kingdom; 3 Biology Program, Faculty of Natural Sciences and Mathematics, Universidad del Rosario, Bogota, Colombia; Indiana University, United States of America

## Abstract

Hybridisation and introgression can dramatically alter the relationships among groups of species, leading to phylogenetic discordance across the genome and between populations. Introgression can also erode species differences over time, but selection against introgression at certain loci acts to maintain postmating species barriers. Theory predicts that species barriers made up of many loci throughout the genome should lead to a broad correlation between introgression and recombination rate, which determines the extent to which selection on deleterious foreign alleles will affect neutral alleles at physically linked loci. Here, we describe the variation in genealogical relationships across the genome among three species of *Heliconius* butterflies: *H*. *melpomene* (*mel*), *H*. *cydno* (*cyd*), and *H*. *timareta* (*tim*), using whole genomes of 92 individuals, and ask whether this variation can be explained by heterogeneous barriers to introgression. We find that species relationships vary predictably at the chromosomal scale. By quantifying recombination rate and admixture proportions, we then show that rates of introgression are predicted by variation in recombination rate. This implies that species barriers are highly polygenic, with selection acting against introgressed alleles across most of the genome. In addition, long chromosomes, which have lower recombination rates, produce stronger barriers on average than short chromosomes. Finally, we find a consistent difference between two species pairs on either side of the Andes, which suggests differences in the architecture of the species barriers. Our findings illustrate how the combined effects of hybridisation, recombination, and natural selection, acting at multitudes of loci over long periods, can dramatically sculpt the phylogenetic relationships among species.

## Author summary

Many species occasionally hybridise and share genetic material with related species. Interspecific gene flow may be counteracted by natural selection at particular ‘barrier loci’. As a result, a pair of species can end up sharing more genetic variation in some parts of their genome than in others, and the tree of relationships in a group of species can differ from one part of the genome to another. We studied relationships and barriers among three species of *Heliconius* butterflies using whole-genome sequences from nine populations. We find that species relationships vary dramatically and predictably across the genome because the species barriers are more porous in genomic regions with higher recombination rates. This occurs because recombination determines how broadly the surrounding genome is affected by a barrier locus. The genome-wide pattern suggests that barrier loci are widespread across the genome. One consequence is that smaller chromosomes, which have higher recombination rates, tend to have weaker species barriers than longer chromosomes. The relationships among populations on small chromosomes therefore tend to be predicted by geography, rather than by which species they belong to. Our work shows how hybridisation, recombination, and selection interact to reshape species’ relationships.

## Introduction

The genealogical relationships among closely related species can be complex, varying across the genome and among individuals. This phylogenetic heterogeneity can be caused both by incomplete lineage sorting (ILS) in ancestral populations and by introgressive hybridisation, causing some parts of the genome to have genealogies that are discordant with the species branching pattern or ‘species tree’. Genome-scale studies have revealed that particular genomic regions such as sex chromosomes and chromosomal inversions can have distinct phylogenetic histories [1–3], possibly reflecting systematic differences in the extent of introgression across the genome. Indeed, the establishment of barriers to introgression in certain parts of the genome is a key part of the speciation process [4–8]. The heterogeneous landscape of species relationships can therefore carry information about the ‘barrier loci’ that contribute to the origin and maintenance of species.

Barrier loci can be associated with extrinsic (imposed by the environment) or intrinsic (affecting viability of fertility) selective pressures, and can act at both prezygotic or postzygotic levels [6,9]. The barriers between closely related subspecies or ecotypes that interbreed frequently are often restricted to just a few loci that contribute to local adaptation, resulting in narrow ‘islands’ of genetic differentiation between populations [1,10–12]. As speciation proceeds, we expect an accumulation of barrier loci, leading to reduced gene flow and more widespread genetic differentiation across the genome [8,13]. Recently, it has become evident that patterns of genomic differentiation between more strongly isolated species are often complex and reflect not only barriers to introgression but also within-species processes that cause variation in effective population size (*N*_*e*_) across the genome, including localised selective sweeps and background selection [14–16]. Relative measures of genetic differentiation, such as the fixation index (*F*_ST_), which are sensitive to variation in *N*_*e*_, therefore provide a poor proxy for the strength of a local barrier [14,15,17]. However, it is possible to largely avoid the confounding effects of positive and background selection by using methods that intrinsically account for heterogeneity in *N*_*e*_ and directly estimate the ‘effective migration rate’ or the level of admixture and how it varies across the genome, either using summary statistics [18] or through model-based inference [19]. Provided that there has been sufficient introgression between the species, regions of the genome in which admixture is reduced can be inferred to have experienced selection against foreign genetic variation.

If species barriers are highly polygenic (made up of many loci) [20] and each locus has only a weak effect on fitness, their individual localised effects on levels of admixture might be difficult to detect, analogous to the difficulties in studying polygenic adaptation more generally [21–23]. Whereas it may not be possible to identify all barrier loci in such a situation, we can test hypotheses about the architecture of barriers by studying genome-wide patterns of admixture. In particular, barriers made up of many loci of small effect are expected to be more porous to introgression where recombination rates are higher. Foreign chromosomes that enter a population through hybridisation and backcrossing will be more rapidly broken down over subsequent generations in regions with higher recombination rates. This will tend to separate clusters of foreign deleterious alleles, reducing selection against them, and also break their linkage with neutral (or mutually beneficial) foreign alleles at other loci, allowing these to avoid removal by selection [19,24–27]. A correlation between the recombination rate and the inferred rate of effective migration has been observed between subspecies of house mice [28], subspecies of *Mimulus* monkeyflowers [19,29], in hybrid populations of swordtail fishes [30], and even between humans and Neanderthals [30,31], suggesting that loci experiencing selection against introgression among close relatives can be widespread in the genome. Therefore, a combination of extensive hybridisation and polygenic barrier loci could theoretically produce predictable large-scale heterogeneity in phylogenetic relationships across the genome, with regions of higher recombination rate showing greater discordance with the species tree. Discordance caused simply by ILS in ancestral populations is also expected to be elevated in regions of higher recombination rate, due to their typically larger *N*_e_ [32]. However, the effects of introgression should be distinguishable in that particular discordant topologies—those that group hybridising species pairs—should be overrepresented.

We explored species relationships and barriers to introgression among species of *Heliconius* butterflies. Many *Heliconius* species are divided into geographically distinct ‘races’ with distinct warning patterns, which signal their distastefulness to local predators. Selection favouring locally recognised warning patterns maintains narrow islands of divergence at a few wing-patterning loci between otherwise genetically similar races [1,10,33]. However, there are also more strongly differentiated pairs of sympatric species that hybridise rarely and have strong postzygotic barriers, leading to higher genome-wide genetic differentiation [1]. We studied three such species—*H*. *melpomene* (‘*mel*’), *H*. *cydno* (‘*cyd*’), and *H*. *timareta* (‘*tim*’)—which form at least two independent zones of sympatry separated by the Andes mountains. Whereas *mel* is found throughout much of South and Central America, *cyd* is largely restricted to the west of the Andes and the inter-Andean valleys, where it overlaps with the western populations of *mel*, and *tim* occurs only on the eastern slopes of the Andes, where it co-occurs with the eastern populations of *mel*. In addition to strong assortative mating based on chemical cues, along with visual cues in the case of *cyd* and *mel* [34–39], both species pairs show ecological differences as well as partial hybrid sterility [36,37,40–44] (and see [36] for a review). Nevertheless, previous studies have revealed surprisingly pervasive admixture between these species in sympatry, most likely explained by a low rate of ongoing hybridisation over an extended period of time [1,45,46]. There is also considerable heterogeneity in the relationships among these populations across the genome [1]. Adaptive introgression in *Heliconius* is well documented. Mimicry between sympatric races of *mel* and *tim* has been facilitated by exchange of multiple wing-patterning alleles [47,48], and at least one case of introgression between *mel* and *cyd* has allowed the latter to mimic other unpalatable species [49]. However, the extent to which introgression among these species might be selected against remains unclear.

Using 92 whole-genome sequences, we asked whether the heterogeneous relationships observed among these species reflect the influence of polygenic barriers to introgression that vary in their strength across the genome. Then, taking advantage of high-resolution linkage maps for these species [50], we show that admixture is correlated with recombination rate, consistent with polygenic species barriers leading to widespread selection against introgression. This selection also explains broader variation in admixture at the chromosomal scale. Overall, our results highlight the pervasive role of natural selection in shaping the ancestry of hybridising species.

## Results

### Population structure

**Fig 1:**
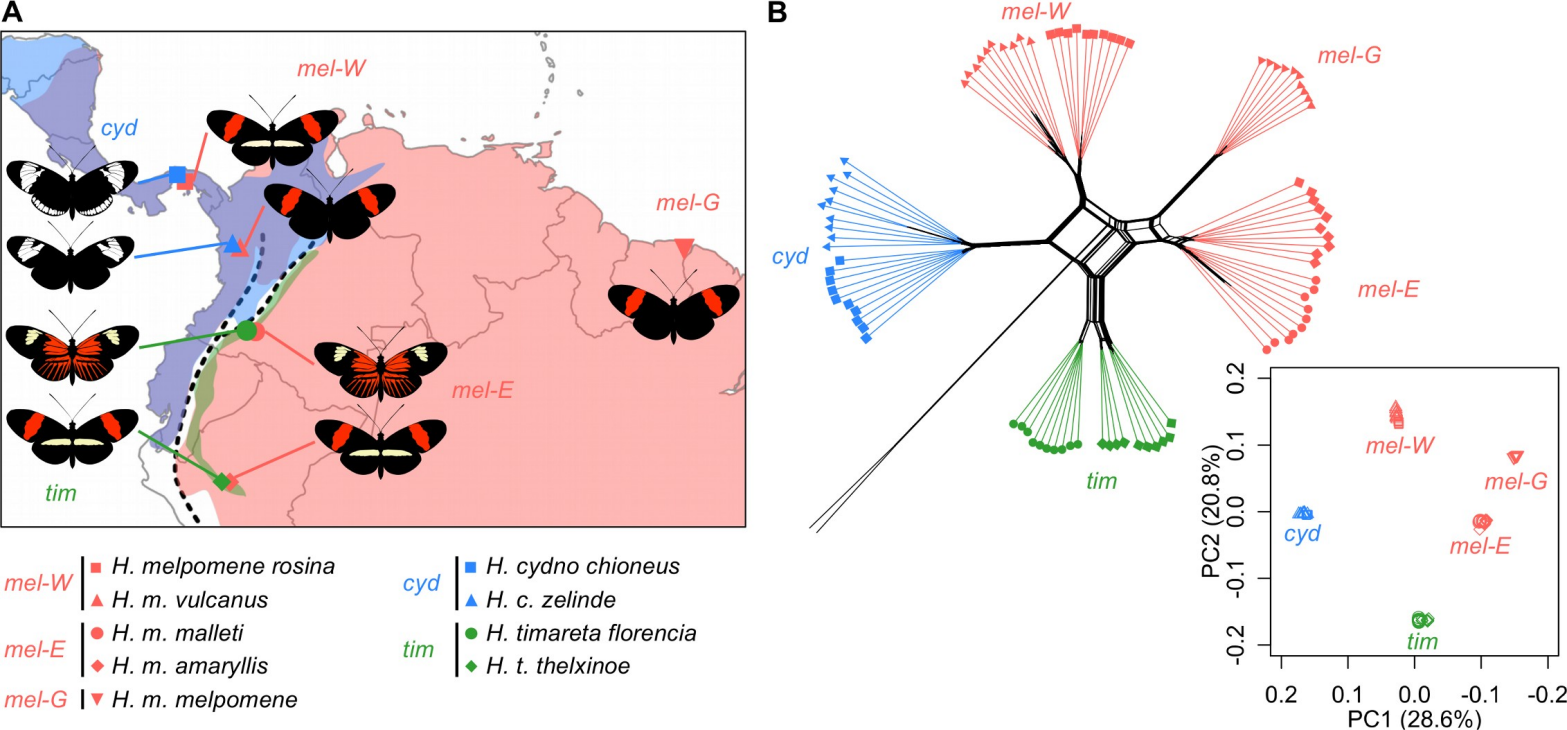
The three species are clearly distinct but show reticulate relationships. (A) Sampling locations of the nine races from three species included in this study (coordinates provided in S1 Table). Species ranges [53] for *mel*, *cyd*, and *tim* are indicated by red, blue, and green shading, respectively. The dashed line indicates the central part of the Andean mountains. While *mel* occurs on both sides of the Andes, *cyd* is restricted to the west of the Andes, and *tim* is restricted to the east of the Andes. Map generated using R package ‘maps’ [54]. (B) Distance-based phylogenetic network and plot of principle components 1 and 2 based on genome-wide SNPs (data deposited in the Dryad repository [55]). Colours and symbols are as in panel A. Principal components 1 and 2 differentiate both *cyd* and *tim* from three *mel* populations but do not separate the two sampled races of each species on either side of the Andes (e.g., *H*. *melpomene rosina* from Panama and *H*. *m*. *vulcanus* from Colombia form a single cluster, which we have therefore considered as a single population, *mel*-W). The same is true for the two populations making up *mel*-E, *cyd*, and *tim*. *cyd*, *H*. *cydno*; *mel*, *H*. *melpomene*; *mel*-E, eastern races of *mel*; *mel*-G, French Guiana *mel*; *mel*-W, western races of *mel*; PC, principal component; SNP, single-nucleotide polymorphism; *tim*, *H*. *timareta*.

We analysed whole-genome sequence data from 92 butterflies representing nine populations from the three focal species, *mel* (5 populations or ‘races’, 10 individuals each), *cyd* (two races, 10 individuals each), and *tim* (two races, 10 individuals each), along with two individuals from an outgroup species *H*. *numata* (‘*num*’) (S1 Table). Our sampling included four regions of sympatry: two on the west of the Andes where *cyd* co-occurs with western races of *mel* (hereafter *mel*-W) and two on the eastern slopes of the Andes where *tim* co-occurs with eastern races of *mel* (hereafter *mel*-E), as well as an allopatric population from French Guiana (hereafter *mel*-G) (Fig 1A). Principal components analysis (PCA) and a phylogenetic network based on whole-genome single-nucleotide polymorphism (SNP) data show clear distinctions between the three species, as well as between *mel*-W, *mel*-E, and *mel*-G (Fig 1B). By contrast, pairs of races of the same species from the same broad geographic area (i.e., west of the Andes, east of the Andes, or French Guiana) are not clearly distinct in the PCA, indicating nearly panmictic populations in each species in each area, despite variation at a few wing-patterning loci, as shown previously [51,52]. The tight clustering and lack of intermediate individuals in the PCA indicates that none of the sampled individuals result from recent hybridisation, consistent with observations that hybridisation is very rare on a per-individual basis. However, large reticulations in the network are consistent with extensive introgression, which has probably occurred gradually through rare hybridisation events spread across millions of generations [1]. These results therefore highlight the contrast between the strong barriers that exist between species—even in sympatry—and the continuity that exists within species, with the Andes mountains and wide Amazon basin presenting the only major sources of discontinuity among sampled populations of the same species [51,52].

### Topology weighting reveals phylogenetic discordance consistent with extensive introgression

**Fig 2:**
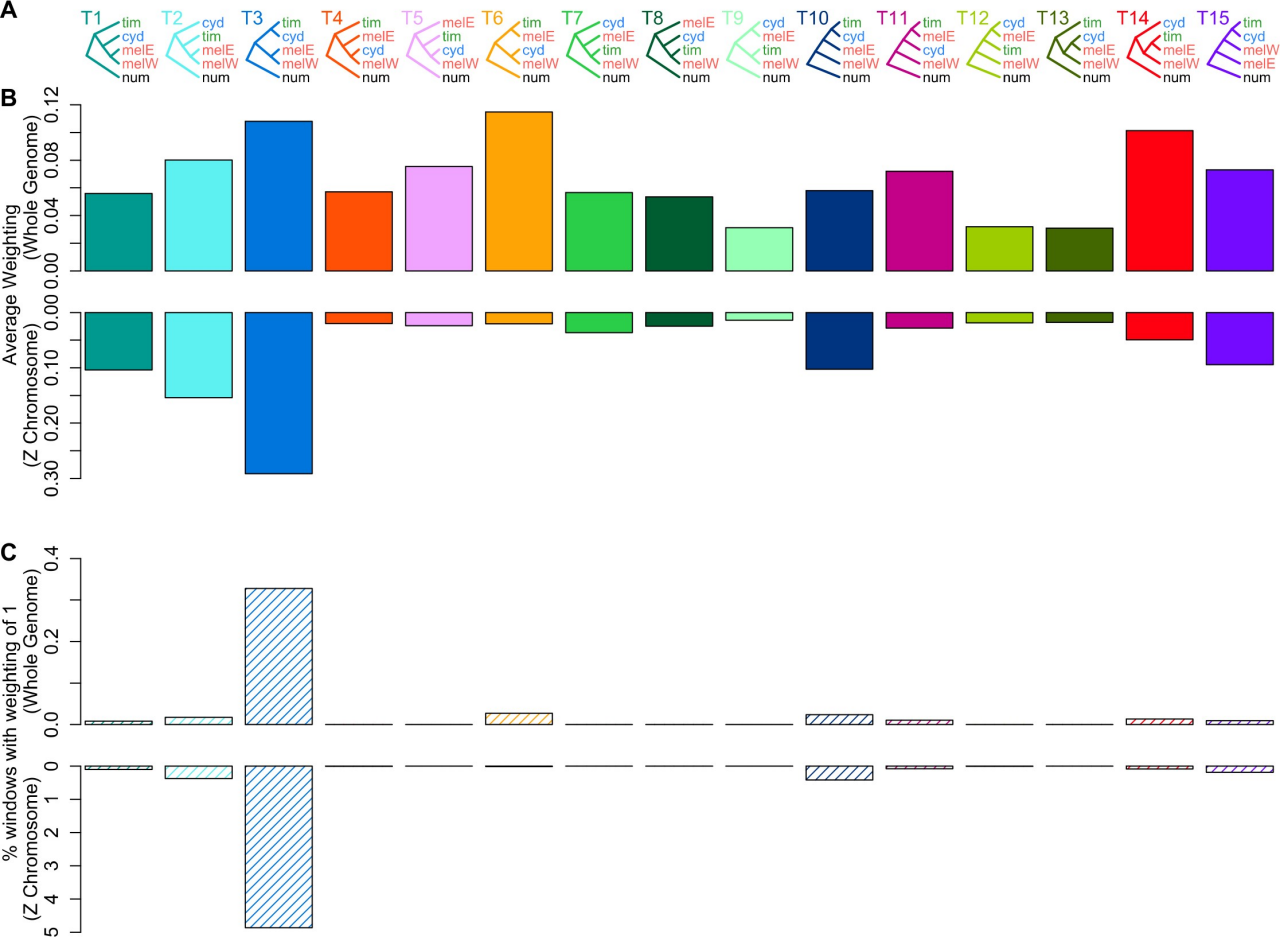
Topology weighting reveals widespread phylogenetic discordance consistent with introgression on autosomes. (A) The 15 possible rooted topologies representing relationships among 4 ingroup taxa: *cyd*, *tim*, *mel-*W, and *mel-*E. (B) Topology weightings for each of the 15 topologies in panel A, averaged across all 50 SNP windows genome wide (upper) and for the Z chromosome only (lower). (C) The percentage of windows with a weighting of 1 (i.e., maximal weighting for a single topology), indicating complete monophyly, genome wide (upper) or for the Z chromosome only (lower). Data deposited in the Dryad repository [55]. *cyd*, *H*. *cydno*; *mel*, *H*. *melpomene*; *mel-*E, eastern races of *mel*; *mel-*W, western races of *mel*; *num*, *H*. *numata*; SNP, single-nucleotide polymorphism; *tim*, *H*. *timareta*.

We explored species relationships across the genome using Twisst [56], which quantifies the frequency (or ‘weighting’) of alternative topological relationships among all sampled individuals in narrow windows of 50 SNPs each. Consistent with previous results, topology weighting indicates that large-scale introgression has shaped the relationships among these species. Examples of local genealogies and their corresponding topology weightings are shown in S1 Fig. All 15 possible rooted topologies that describe the relationship between *cyd*, *tim*, *mel*-W, and *mel*-E (rooted with *num* as the outgroup) are represented at considerable levels across the genome (Fig 2). Moreover, fewer than 0.5% of windows have completely sorted genealogies (i.e., all groups cluster according to a single topology, resulting in a weighting of 1, see S1 Fig for an example) (Fig 2C). Coalescent simulations using an appropriate split time (approximately 1.5 million years ago [45,46,57]) and population size (2 million [52]) show that, in the absence of introgression, we would expect far less phylogenetic discordance and more complete lineage sorting (more windows with a weighting of 1 for a single topology) than seen here, unless the population sizes were much larger (S2 Fig). However, the addition of moderate gene flow between sympatric pairs produces levels of discordance and lineage sorting similar to our empirical results (S2 Fig). The two most common topologies across the genome are T3 and T6, which differ entirely in the relationships among the ingroup taxa (Fig 2A). T3 matches the expected species branching order, in which *cyd* and *tim* are sister species and *mel*-W groups with *mel*-E ([*cyd*, *tim*], [*mel*-W, *mel*-E]) [51,58]. We refer to this as the ‘species topology’. T6, by contrast, groups populations by geography: *cyd* with *mel*-W, and *tim* with *mel*-E ([*cyd*, *mel*-W], [*tim*, *mel*-E]). We refer to this as the ‘geography topology’. We therefore hypothesise that the history of these species can be modelled as a branching process following the species topology, with considerable introgressive hybridisation beginning at some point after the species diverged that increases the rate of coalescence between sympatric populations from distinct species, as in the geography topology. Although the geography topology has a slightly higher average weighting, the species topology occurs far more frequently with a weighting of 1 than any other topology (Fig 2C). In other words, the only pairs of populations that consistently show complete monophyletic clustering are *mel*-W with *mel*-E and *cyd* with *tim* (for an example, see S1 Fig). Our simulations agree that, although extensive introgression can also produce high rates of monophyly, this level of monophyly between allopatric populations is only expected if they are sister taxa (S2 Fig), thus supporting T3 as the true species branching pattern. The species topology also agrees with ecological trends such as host plant use, as well as both larval and adult morphology, which support a sister relationship of *cyd* and *tim* [59,60].

Topology weightings for the Z chromosome are dramatically different from the genome-wide averages (Fig 2B). There is much less discordance, and the species topology has by far the highest weighting. Moreover, the geography topology and others consistent with introgression have comparatively low weightings. The contrasting abundance of discordant topologies on the autosomes could be explained not only by much higher levels of introgression affecting the autosomes, as shown previously [1], but also by their larger *N*_e_, and resulting slower rate of lineage sorting compared to the Z. However, as shown by our simulations (S2 Fig), only extensive introgression can explain the strong skews in the topology weightings on the autosomes—with the geography topology (T6) being the most abundant—whereas others such as T9, which groups allopatric nonsister taxa, is among the least abundant genome wide. Similarly, the third and fourth most highly weighted topologies across autosomes (T14 and T5) both group one pair of sympatric taxa (*mel*-E with *tim* and *mel*-W with *cyd*, respectively) and otherwise match the species topology (Fig 2B). Taken together, these patterns are all consistent with extensive postspeciation introgression between sympatric pairs, with a strong reduction in introgression on the Z chromosome.

Our sampling design allows us to make inferences about biases in the direction of gene flow. T14—which has *tim* nested within the *mel* clade, suggesting introgression predominantly from *mel*-E into *tim* (Fig 2B)—is the third most abundant topology genome wide and has a higher weighting than T11, which implies gene flow in the opposite direction. A bias towards introgression into *tim* would be expected given the much smaller range and lower *N*_*e*_ of *tim*, which also has the lowest nucleotide diversity of all the taxa studied [1]. Hybrids that backcross into *tim* will therefore provide a larger relative contribution to the gene pool than those that backcross into *mel*. Likewise, T5 is more abundant than T4, suggesting that most introgression in the west of the Andes has been from *cyd* into *mel*-W (Fig 2B). This direction was also inferred to be the most likely in a previous study using coalescent modelling [46] and is consistent with the fact that F1 hybrids show mate preference for *mel* in experiments with Panama populations [34]. Our simulations, in which the direction of introgression was biased in both cases, also show similar imbalances in the weightings for the same topologies.

### Species relationships vary predictably across the autosomes

**Fig 3:**
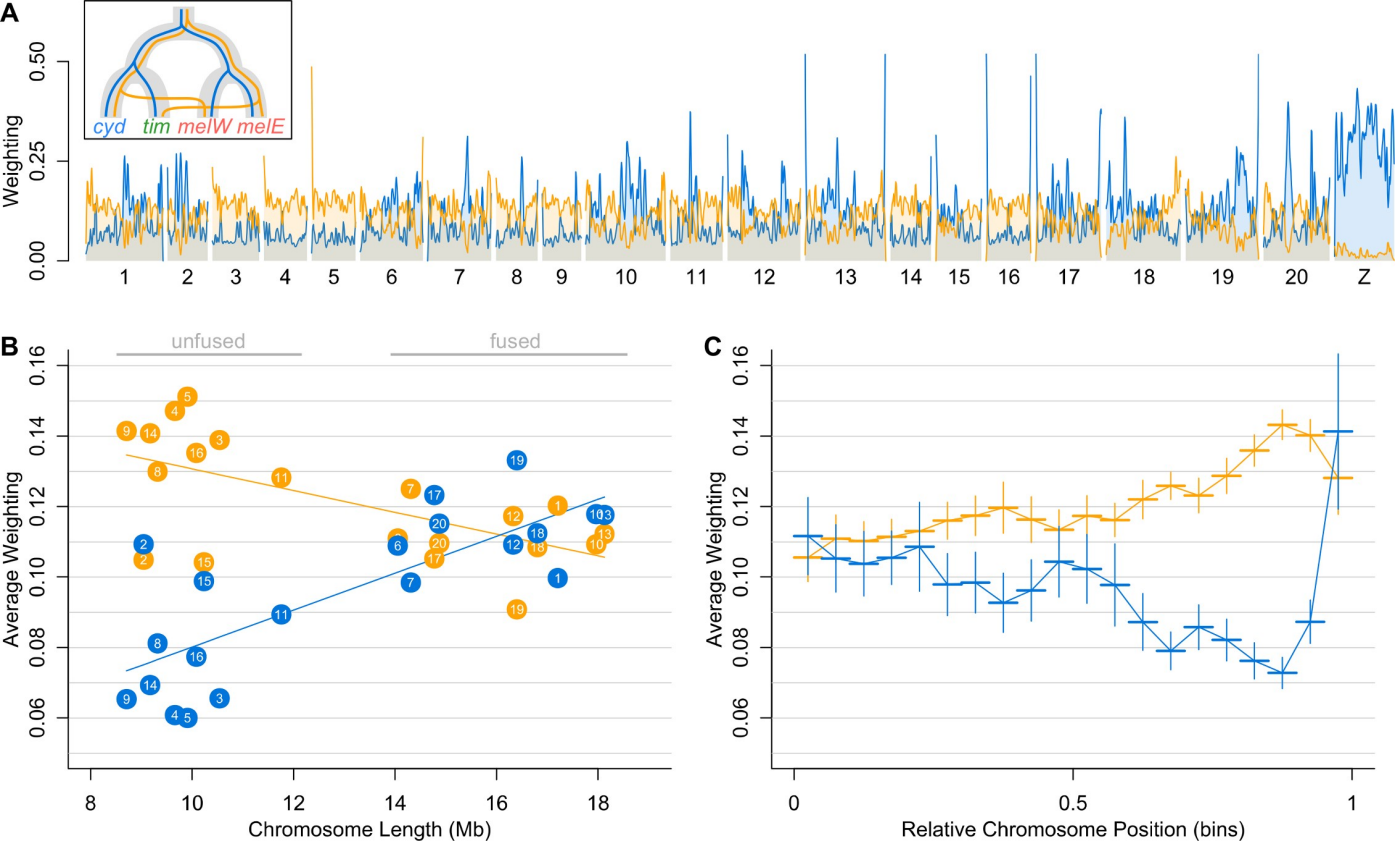
Species relationships vary consistently both among and within chromosomes. (A) Weightings for the ‘species’ (T3; blue) and ‘geography’ (T6; yellow) topologies plotted across the 21 chromosomes and smoothed as a locally weighted average (loess span = 1 Mb). See S3 Fig for a detailed plot without smoothing. Note that in the insert, the indicated direction of introgression in T6, from *cyd* into *mel*-W and from *mel*-E into *tim*, reflects our inferred predominant direction, even though introgression is thought to be bidirectional. (B) The average weighting for the same two topologies (colours as in panel A) for each of the 20 autosomes, plotted against the physical length of the chromosome. (C) Average weightings for the same two topologies (colours as in panels A and B) binned according to their relative chromosome position, from the centre (0) to the periphery (1). Each bin represents 5% of the chromosome arm, with the range indicated by a horizontal line. Vertical lines indicate ±1 SE. All plotted data deposited in the Dryad repository [55]. *cyd*, *H*. *cydno*; *mel*, *H*. *melpomene*; *mel-*E, eastern races of *mel*; *mel-*W, western races of *mel*; *tim*, *H*. *timareta*.

In addition to the difference between autosomes and the Z chromosome, topology weightings vary considerably among and within the autosomes. To highlight this heterogeneity, we first focus on the two most abundant patterns of relatedness: the species and geography topologies (Fig 3A). The species topology has the highest weighting in narrow peaks on some of the autosomes, whereas elsewhere the geography topology has the higher weighting. In other words, throughout large parts of the genome, samples of *mel*-W and *mel*-E tend to be more closely related to their respective sympatric counterparts, *cyd* and *tim*, than to one another. However, the species topology tends to occur in sharp peaks, which frequently have a weighting approaching 1, as discussed above (S3 Fig; note that these narrow peaks are not visible in Fig 3A due to smoothing).

There are strong trends in the abundance of the species and geography topologies across the 20 autosomes. All species in this clade have 10 short and 10 long autosomes. The latter formed through 10 independent fusions in the ancestor of *Heliconius*, which had 30 autosomes [61,62]. The species topology is less abundant on the 10 short autosomes compared to the 10 long, fused autosomes, and there is a fairly linear increase in its weighting with chromosome length (Fig 3B). By contrast, the geography topology shows decreasing abundance with chromosome length and tends to be far more abundant on the short chromosomes. There is also a fairly consistent within-chromosome trend, with higher weightings for the geography topology and lower weightings for the species topology towards the outer third of the chromosomes compared to the chromosome centres (Fig 3C). This reverses in the outer 5% of chromosomes, where the species topology is again strongly supported.

The above trends might be partly explained if ILS in ancestral populations was more common on short chromosomes and away from the centres and very ends of chromosomes, leading to increased discordance in these regions. Indeed, we previously found a negative correlation between chromosome length and *N*_e_ in *mel* [52]. However, the trends shown by other topologies suggest that these patterns also reflect variation in the extent of introgression between genomic regions. The 5 topologies that group allopatric pairs and therefore likely reflect discordance due to ancestral ILS alone (i.e., T7, T8, T9, T12, and T13) show only weak relationships with chromosome length, and no clear pattern within chromosomes (S4 Fig). Furthermore, T14, which is consistent with introgression between *mel*-E and *tim*, is less abundant than the species topology (T3) on long chromosomes and at chromosome centres but is more abundant on short chromosomes and in chromosome peripheries (S4 Fig). Such a switch in rank is not expected if the short chromosomes and peripheries simply experience more ancestral ILS, but it is consistent with differences in the extent of introgression between short and long chromosomes and between centres and peripheries. T5, which is consistent with introgression between *cyd* and *mel*-W, does not show any clear relationship with chromosome length or relative chromosome position (S4 Fig). This implies that there may be less consistent variation in the extent of introgression between *cyd* and *mel*-W in different regions. However, T4, which reflects introgression between the same pair but in the opposite direction (from *mel*-W into *cyd*) does show weak patterns similar to the geography topology. Overall, topology weighting reveals quantitative variation in species relationships both within and among chromosomes that are consistent with heterogeneity in the level of introgression across the genome. However, topology weighting does not explicitly distinguish between introgression and shared ancestral variation. We therefore set out to explicitly test the hypotheses that (1) there is heterogeneity in the level of admixture across the genome and (2) that this heterogeneity can be explained by variation in the strength of selection against introgression.

### Heterogeneous admixture suggests variable selection against introgression

We used the summary statistic *f*_d_ [18] to quantify admixture separately between *cyd* and *mel*-W and between *tim* and *mel*-E. This approach also measures an excess of genealogical clustering of sympatric nonsister taxa. However, *f*_d_ provides a normalised measure that is approximately proportional to the effective migration rate [18]. Building on previous work, we first investigated the degree to which *f*_d_ might be influenced by variation in *N*_*e*_ across the genome. *N*_*e*_ tends to be reduced in regions of reduced recombination rate due to linked selection. By means of simulations, we find that, across a large range of realistic population sizes, *f*_*d*_ is a reliable estimator of admixture. Furthermore, *f*_d_ outperforms the commonly used divergence statistics *F*_ST_ and *d*_XY_, which are both highly sensitive to *N*_*e*_ (S5 Fig). When population sizes are very large, *f*_d_ tends to underestimate the true level of admixture. This is caused by a loss of information when population sizes are large relative to the split times: the lack of lineage sorting means that there is insufficient information available to accurately quantify admixture. The population sizes for which this is relevant are at the upper end of estimates for these species [52]. Moreover, this error would cause a conservative bias in our results, as we expect reduced admixture in low-recombination regions, where *N*_e_ is expected to be the smallest. Most important for our subsequent analysis, high background selection in regions of low recombination—which is known to influence measures such as *F*_ST_—is not likely to strongly bias our estimates using *f*_d_. We therefore conclude that *f*_d_ provides a suitable, albeit conservative, measure to test the hypothesis that species barriers are enhanced in regions of reduced recombination rate.

Computation of *f*_d_ requires the use of a ‘control’ population that is ideally allopatric and unaffected by introgression. To confirm the robustness of our results, we computed *f*_*d*_ with several different sets of populations, varying the control population, as well as splitting or joining each of *cyd*, *tim*, *mel*-W, and *mel*-E into their two constituent subpopulations (S6 Fig).

**Fig 4:**
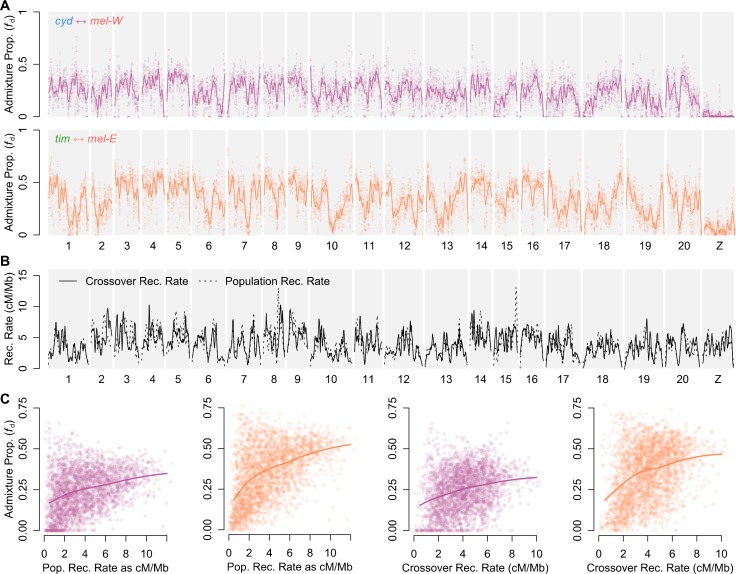
Admixture proportions are correlated with recombination rate. (A) Estimated admixture proportions (*f*_d_) between *cyd* and *mel-*W (upper) and between *tim* and *mel-*E (lower) plotted across all 21 chromosomes in 100 kb windows, sliding in increments of 20 kb. A locally weighted average (loess span = 2 Mb) is included. Results shown are for population Sets 1 and 4 of S6 Fig. See S7 and S8 Figs for more detailed plots. (B) Recombination rate estimated from the crossover rate in linkage maps (solid line) and ρ averaged across the four populations considered and plotted as a locally weighted average (loess span = 2 Mb) (dashed line). See S9 Fig for a more detailed plot. (C) Admixture proportions for *cyd* and *mel-*W (first and third plot) and *tim* and *mel-*E (second and fourth plot) for nonoverlapping 100 kb windows plotted against ρ (first two plots) and crossover recombination rate (last two plots). Solid lines indicate the locally weighted average (loess span = 0.75). All plotted data deposited in the Dryad repository [55]. ρ, population recombination rate; *cyd*, *H*. *cydno*; *mel-*E, eastern races of *mel*; *mel*, *H*. *melpomene*; *mel-*W, western races of *mel*; *tim*, *H*. *timareta*.

Patterns of admixture estimated by *f*_d_ show considerable heterogeneity across the genome (Figs 4A and S7 and S8). As expected, admixture is minimal across the Z chromosome in both pairs, indicating a strong barrier to introgression. There is also heterogeneity in admixture proportion across the autosomes. This is most striking between *tim* and *mel*-*E*, where some regions exhibit deep troughs, implying strong, localised species barriers. Some of this heterogeneity likely reflects individual barrier loci of large effect. Indeed, the known wing-patterning loci provide a useful example. The pattern differences between *cyd* and *mel*-W are determined by regulatory modules around 3 major genes: *wnt-A* (Chromosome 10), *cortex* (Chromosome 15), and *optix* (Chromosome 18) [49,63–67]. These probably act as strong barriers to introgression between *cyd* and *mel*-W, due to increased predation against hybrids with intermediate wing patterns [43]. By contrast, the shared wing patterns of *tim* and *mel*-E are thought to result from adaptive introgression of wing-patterning alleles. As expected, there is a strong reduction in admixture between *cyd* and *mel*-W in the vicinity of all 3 genes (S7 Fig), while there are peaks of admixture between the comimetic *tim* and *mel*-*E* populations in the corresponding regions (S8 Fig).

### Admixture proportions are correlated with recombination rate

We hypothesised that many loci across the genome contribute to the species barriers, which leads to the expectation that the level of admixture will be correlated with the recombination rate [19]. We quantified variation in recombination rate across the genome using high-resolution linkage maps (based on 963 offspring in total) [50] as well as using LDHelmet [68], which estimates the population recombination rate (ρ) based on linkage disequilibrium (LD) in the genomic data from natural populations. On a broad scale, the map-based estimates are highly concordant with the population-based estimates, and the latter are also strongly conserved across the different species (Figs 4B and S9). There is considerable variation in recombination rate across the genome, allowing us to investigate whether admixture proportions are correlated with recombination rate.

There is a strong positive and nonlinear relationship between admixture proportion and recombination rate in both species pairs (Figs 4C and S11). Correlations between *f*_d_ and both the ρ and crossover recombination rate are highly significant, even after thinning windows to reduce effects of serial correlation (S2 Table). Strong reductions in admixture, implying barriers to introgression, are concentrated in genomic regions in which recombination rates are below 2 cM/Mb. However, there is also more variability in admixture proportions in these low-recombination regions, with some showing high estimated levels of admixture (Fig 4C). This might imply that some regions do not harbour loci that contribute to the species barrier, although the variance in admixture proportions may also be increased in low-recombination regions due to increased genetic drift resulting from enhanced linked selection. The correlations persist when considering only regions of intermediate recombination rate (2–8 cM/Mb) (S2 Table). The relationship between admixture and recombination rate is stronger in the *tim* and *mel*-E pair, implying that the more heterogeneous pattern of admixture across the genome between this pair is more consistent with a model in which barrier loci are widespread and recombination rate modulates the strength of the barrier to introgression.

Admixture proportions are less well predicted by the map-based estimates of crossover recombination rate compared to the inferred ρ (Fig 4C and S2 Table). This probably partly reflects inaccuracy in fine-scale recombination rate estimated from the linkage maps. However, it may also be that ρ (= 4*N*_*e*_*r*) provides a more meaningful predictor for the admixture proportion, as it is a composite of the per-generation recombination rate (*r*) and local *N*_e_. Due to linked selection, parts of the genome with a low recombination rate and a high density of selected sites are expected to have locally reduced *N*_*e*_ and therefore reduced ρ. Indeed, ρ is strongly negatively correlated with the proportion of coding sequence per window (referred to as ‘gene density’ hereafter) (Spearman’s rho = −0.681; *p* < 0.001; S10 Fig). However, there is also a weaker but significant negative relationship between gene density and the crossover recombination rate (Spearman’s rho = −0.248; *p* < 0.0001; S10 Fig). This implies that linked selection in regions of low recombination rate may be further enhanced by a higher density of selected loci. As the conditions that enhance linked selection are the same as those expected to strengthen barriers to introgression (i.e., a high ratio of selected loci relative to the recombination rate, also called the ‘selection density’ [19]), it is to be expected that ρ would provide a better predictor of barrier strength and therefore admixture proportion. As expected, there is a negative relationship between admixture proportion and gene density (S12 Fig). However, the fact that regions with a high gene density also tend to have lower recombination rates makes it difficult to determine whether such regions harbour a higher physical density of barrier loci, but this seems likely given the arguments above.

The correlation between admixture and recombination rate remains clear when individual chromosomes are considered separately, with 18 and 20 of the 21 chromosomes showing a significant correlation for *mel*-W/*cyd* and *mel*-E/*tim*, respectively (S13 Fig). This further highlights the genome-wide nature of this trend. The above trends are also robust to using different allopatric ‘control’ populations when estimating admixture proportions (S11 and S12 Figs), with the exception that using very closely related control populations leads to very low estimated rates of admixture, for which the relationships with recombination rate and gene density are not clear due to the large number of windows at which the estimated admixture proportion is 0. See S11 and S12 Figs for details.

### Reduced admixture on longer chromosomes

**Fig 5:**
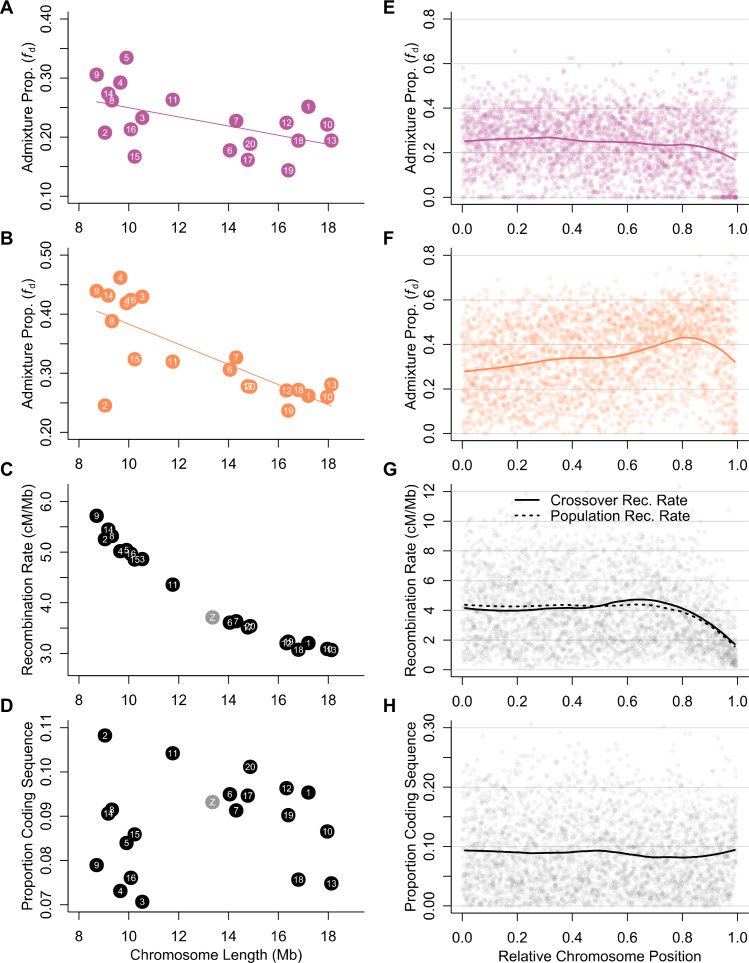
Variation in admixture proportions among and within chromosomes is explained by recombination rate and distance from chromosome centres. (A, B) Estimated admixture proportions (*f*_d_) between *cyd* and *mel-*W (panel A) and between *tim* and *mel-*E (panel B) for each chromosome plotted against chromosome length. Population Sets 1 and 4 were used for these analyses (see S6 Fig). (C) Crossover recombination rate for each chromosome plotted against chromosome length. (D) Proportion of coding sequence for each chromosome plotted against chromosome length. (E, F) Estimated admixture proportions (*f*_d_) between *cyd* and *mel-*W (panel E) and between *tim* and *mel-*E (panel F) for 100 kb windows plotted against relative chromosome position. A locally weighted average (loess span = 0.25) is included. (G) Crossover recombination rate plotted against relative chromosome position. A locally weighted average is included, along with the corresponding line for ρ. (H) Proportion of coding sequence per 100 kb window plotted against relative chromosome position, again with a locally weighted average shown. All plotted data deposited in the Dryad repository [55]. ρ, population recombination rate; *cyd*, *H*. *cydno*; *mel-*E, eastern races of *mel*; *mel*, *H*. *melpomene*; *mel-*W, western races of *mel*; *tim*, *H*. *timareta*.

Average chromosomal admixture proportions are negatively correlated with chromosome length (Fig 5A and 5B). This is expected given the extremely strong negative correlation between physical chromosome length (in base pairs) and average recombination rate (Spearman’s rho = −0.98; *p* = 4.6 × 10^−6^) (Fig 5C). By contrast, there is no clear relationship between chromosome length and gene density (Spearman’s rho = 0.058; *p* = 0.8) (Fig 5D). The broadly enhanced barrier to introgression on long chromosomes is therefore more consistent with an effect of increased linkage, rather than an increased density of barrier loci. As in the trends above, the correlation with chromosome length is stronger for admixture between *tim* and *mel*-E (Spearman’s rho = −0.66; *p* = 7 ×10^−4^) than for admixture between *cyd* and *mel*-W (Spearman’s rho = −0.51; *p* = 0.012). Between *tim* and *mel*-E, the shortest chromosomes experience about 50% more admixture than the longest chromosomes, with the exception of Chromosome 2, which has strongly reduced admixture compared to other short chromosomes with similarly high recombination rates. This might reflect a higher density of barrier loci on this chromosome, which seems possible because it also has the highest gene density of all chromosomes (Fig 5D).

Given that the 10 longer chromosomes all arose through fusions in the ancestor of the genus [62], we also investigated whether there was some additional effect of the fusions themselves in causing reduced admixture, apart from the obvious indirect effect of lower average recombination rates on longer chromosomes. There is no consistent trend of reduced admixture in the vicinity of the fusion points themselves (S7 and S8 Figs). We tested for a general difference between fused and unfused chromosomes independent of recombination rate by binning 100 kb windows by local recombination rate and then comparing bins of equivalent recombination rates between fused and unfused chromosomes (S13 Fig). In both species pairs, most bins showed significantly reduced admixture on the fused chromosomes. This suggests that their reduced recombination rate alone may be insufficient to explain the extent of reduction in admixture, implying a higher density of barrier loci on fused chromosomes, despite no significant difference in gene density (Fig 5D). However, the same pattern would be expected if admixture proportions in 100 kb windows are influenced by recombination rates in surrounding regions, so it is difficult to distinguish the effect of lower global recombination rates on longer chromosomes from a difference in the density of barrier loci.

### Variation in admixture along chromosomes

As indicated by topology weighting, there is an effect of position along the chromosome on the proportion of admixture between *mel*-E and *tim*, where admixture increases on average towards the distal region of the chromosome but decreases again at chromosome ends (Fig 5F). This is not seen in the proportion of admixture between *cyd* and *mel*-W (Fig 5E), which appears to show a weak decline moving away from the chromosome centre and a sharper decline at the end. Unlike in many other taxa [27], there is no consistent decrease in recombination rate towards chromosome centres. By contrast, both the crossover recombination rate and ρ show a sharp decrease at the chromosome ends (Fig 5G). Gene density is roughly uniform across chromosomes on average (Fig 5H). Theory predicts that, given a uniform recombination rate and distribution of selected loci, species barriers should weaken towards chromosome ends due to a decrease in the number of linked deleterious alleles on one side of the focal locus (i.e., an ‘edge effect’), leading to increased admixture towards chromosome ends [25]. Therefore, the different trends seen in the two species pairs might imply a different balance between this edge effect, which should weaken species barriers, and reduced recombination, which should strengthen them.

## Discussion

Introgression effectively acts to rewrite the evolutionary history of the genome. Genome-scale data have revealed that the extent of introgression in some species may be far greater than previously imagined [1,2,69,70]. Despite the strong behavioural isolation among the three species studied here, we find that relationships among them vary dramatically and predictably across the genome, and that in some parts, introgression has overwhelmed the genealogical footprints of the original population branching pattern. Similar dramatic heterogeneity in species relationships has been described in several other taxa [2,71]. For example, introgression among some *Anopheles* mosquitos has almost entirely eliminated the signal of the original species branching events on autosomes [2]. In the present study, by analysing genomes from multiple samples per population, we show that *Heliconius* species relationships vary quantitatively within and among chromosomes. Our main finding is that this variation in species relationships is predictable and can be partly explained by quantitative variation in the strength of the selective barrier to introgression, which depends on the local recombination rate. Our findings therefore show how hybridisation and natural selection act in combination to shape the tree of life.

There has been considerable interest in making inferences about species barriers from the genomic landscape of divergence between hybridising species, based on the idea that selection should resist genetic homogenisation by gene flow at barrier loci. However, there has also been an increasing realisation that patterns of differentiation and divergence can be influenced by unrelated factors, such as linked selection acting within species [15,72–74]. One effect of these confounding factors is that relative measures of divergence, such as *F*_ST_, can show elevated values in regions of the genome that experience stronger linked selection, even if there is no reduction in effective migration in such regions, and indeed even when there is no gene flow at all [16]. In other words, the observation of increased *F*_ST_ in regions of the genome with reduced recombination rate is not necessarily informative about the architecture of species barriers. This is particularly problematic when considering species that hybridise rarely, as the contribution of gene flow to patterns of differentiation may be small compared to that of within-species linked selection. Previous analyses of these *Heliconius* species revealed a highly heterogeneous pattern of *F*_ST_, in which even known wing-patterning loci that have a major impact on hybrid fitness are not particularly prominent [1]. Until now, it has not been known whether barrier loci are indeed widespread throughout the genome in these species. The availability of new approaches that avoid the shortfalls of *F*_ST_ by directly inferring the admixture proportion or effective migration rate [18,19,75] now make it possible to examine the relationship between recombination and admixture, revealing the architecture of species barriers.

Our results suggest that there are highly polygenic barriers that maintain these *Heliconius* species. There is a strong correlation between admixture and recombination, both genome wide and on individual chromosomes. Windows in regions of high recombination rate (>5 cM/Mb) almost invariably show increased levels of admixture, whereas windows showing reduced admixture are concentrated in parts of the genome with low recombination rates (<2 cM/Mb). This is consistent with theoretical expectations that widespread barrier loci will cause a stronger localised reduction in introgression in regions of lower recombination rate, due to increased linkage among selected loci, and between selected and neutral loci [19,24–26,29,76]. Some windows in regions of low recombination nevertheless show high levels of admixture. This may indicate that barrier loci, although abundant, are not ubiquitously distributed across the genome. However, we also expect increased variance in levels of admixture in these low-recombination regions due to increased genetic drift, which will be compounded by the reduced independence among sites in the 100 kb windows. This increased variance does not explain the positive relationship between admixture and recombination, however, and the relationship persists when the regions with the lowest recombination rates are excluded from the analysis.

Selection against introgression also produces a global pattern of decreasing admixture with chromosome length. Long chromosomes, which have similar gene density but lower per-base recombination rates than short chromosomes, form stronger barriers to introgression on average. The 10 long chromosomes that resulted from fusions in the ancestor of *Heliconius* showed more resistance to introgression than unfused chromosomes even in regions of equivalent recombination rate. This might indicate an average higher density of barrier loci on the fused chromosomes. However, it could also be explained if the effects of linked barrier loci extend beyond the range of the 100 kb windows that we analysed, such that introgression in any given window is affected by recombination rates in surrounding regions, which will typically be lower on the longer fused chromosomes. This could be resolved in the future by explicitly modelling the relationship between recombination and selection against introgression at the whole-chromosome scale.

Our experimental approach does not consider the possible effects of changes in the recombination landscape between the species [77]. Comparison of linkage maps [50] and population recombination rates suggest that there is minimal large-scale change in recombination rates among these species. Nonetheless, even changes to the recombination landscape over longer time periods are important because they determine which parts of the genome are likely to be involved in forming future species barriers. For example, the 10 fusions that produced longer chromosomes with lower recombination rates in *Heliconius* occurred after divergence from its sister genus, *Euides* [62], implying that species barriers in the latter group might follow a very different landscape.

It is likely that *Heliconius* species barriers are also stronger in gene-rich regions, due to an increased density of barrier loci. While we did find a weak trend of reduced admixture in gene-rich regions, this is difficult to interpret because the recombination rate is also lower in gene-rich regions in these species. An additional factor that could influence our conclusions is the fact that barrier loci may not be expected to accumulate randomly across the genome. Some models predict that, under a scenario of ecological divergence in the face of gene flow, the accumulation of barrier loci may be clustered [26,78]. This could increase the correlation between admixture and recombination and perhaps lead to overestimation of the density of barrier loci. Clustering could theoretically be further enhanced by genomic rearrangements that bring together loci involved in local adaptation [79] or that physically suppress recombination in hybrids. However, we have previously found that there are no major inversions or regions of suppressed recombination in hybrids that maintain barriers between *mel* and *cyd* [50]. Nonetheless, the lack of information about the distribution of barrier loci and their effect sizes means that it is currently not possible to estimate the number of loci involved, except that it is probably very large.

In agreement with previous findings, we find that barriers to introgression are far stronger across the Z chromosome compared to autosomes. Enhanced barriers to introgression on sex chromosomes have been observed in genomic studies of a range of taxa with both XY and ZW systems [1–3,80]. This has been attributed to a more rapid build-up of incompatibilities due to hemizygosity and a key role played by sex chromosomes in reproduction and fertility. Comparing genetic differentiation on sex chromosomes to autosomes can be complicated by their reduced *N*_e_, and this can be further confounded by changes in population size, which can affect sex chromosomes differently [81]. However, our simulations show that these factors cannot explain the reduction in admixture detected here using *f*_d_. In these *Heliconius* species, hybrid female sterility is associated with 1 or more loci on the Z chromosome [42,82,83]. The observed reduction of admixture across the Z chromosome must result from selection against foreign Z chromosome alleles in backcross progeny and their descendants, such that there are opportunities for independent assortment of chromosomes prior to selection. Segregation of sterility in backcrosses has indeed been observed in crossing experiments [42,83,84]. This also means that there are opportunities for recombination before selection. The fairly even reduction in admixture we observe across most of the chromosome is therefore perhaps surprising and implies that there are multiple barrier loci spread across the Z chromosome. A similar pattern of widespread incompatibilities throughout much of the sex chromosome has been shown experimentally between *Drosophila* species [85].

Perhaps the most important trend in our results is that regions of the genome that harbour the strongest species barriers correspond with those showing the greatest phylogenetic concordance with the species tree. Theoretical models show that genealogies of barrier loci might not be expected to conform to the species branching pattern because the selected alleles can arise before lineage sorting is complete [86]. This apparent contradiction may be resolved by the fact that the regions in which species barriers are strongest (low-recombination regions and the Z chromosome) are also those expected to have generally lower *N*_e_ (due to linked selection [87] and hemizogosity, respectively). The resulting increase in the rate of lineage sorting in such regions is expected to decrease discordance, as has been observed in other taxa [32,88]. The strong correspondence between barrier regions and the species tree may therefore be expected. Our results demonstrate that introgression can play a similar, and perhaps more dramatic role, than ILS in driving predictable patterns of phylogenetic discordance across the genome.

The model proposed here of a highly polygenic species barrier between *mel* and its relatives contrasts with previous studies that identified a few major-effect loci that control differences in wing pattern and mate preference between *cyd* and *mel* [43,84]. In particular, a recent study identified 3 major-effect quantitative trait loci (QTL) for assortative mating behaviour, one of which is tightly linked to the *optix* wing-patterning locus [89]. Nonetheless, the presence of a few major QTL affecting premating isolation does not directly contradict our results, because multiple additional behavioural, ecological, and fertility-related barriers are known to act between these species [36,59], and each of these may have a more polygenic basis. Unlike *cyd*, *tim* races have wing patterns that commonly match those of the local *mel* races. Therefore, the large-effect wing-patterning loci do not contribute to the barrier between *tim* and *mel*. This difference might explain why admixture between this pair is more strongly correlated with recombination rate. When barrier loci are weak and dispersed across the genome, admixture proportions should be more strongly predicted by recombination rate than when there are few large-effect barrier loci. Therefore, perhaps counterintuitively, the more heterogeneous pattern of admixture between *tim* and *mel* is in fact more consistent with the model of small-effect barrier loci evenly distributed across the genome. The heterogeneity reflects the underlying heterogenous recombination landscape, rather than a patchy distribution of large-effect barrier loci.

The cause of the more even pattern of admixture between *cyd* and *mel*-W is less clear. One possible explanation is a different architecture of the species barrier, with more epistatic interactions among barrier loci, which might lead to a deviation from theoretical predictions that largely ignore epistasis. Lindtke and Buerkle [90] distinguish between two types of epistatic barrier loci: classic Dobzhansky-Muller incompatibilities (DMIs) that cause reduced fitness in hybrids but can be broken down by recombination in backcross progeny, following the formulation by Dobzhansky [4] and Muller [7], and ‘pathway’ incompatibilities that reduce fitness in recombinant hybrids in which co-adapted alleles become separated. Simulations show that pathway-type incompatibilities can produce pronounced localised barriers to introgression, whereas classic DMIs can have more even, genome-wide effects if selection is strong enough [90]. The large-effect wing-patterning loci, which only contribute to the *cyd*/*mel* barrier, may act as classic DMIs. Hybrids have intermediate wing patterns that do not resemble the recognisable warning patterns of either species, making them roughly twice as vulnerable to predation [43], but backcrossing restores a recognisable warning pattern to a proportion of the progeny. This unique additional barrier between *cyd* and *mel* is likely to be most significant in early-generation backcross hybrids, which is where most selection against introgression is likely to occur [91]. It is therefore possible that the presence of these large-effect incompatibilities could help explain the less heterogeneous landscape of introgression in this species pair, although this requires further investigation.

In conclusion, our findings imply that barrier loci have accumulated rapidly in the 1 to 1.5 million years over which these butterfly species have diverged. This joins a growing number of examples showing that selection against introgression between fairly young species can be pervasive across the genome [19,27–31]. Further work is still required to determine the generality of these trends and to account for complications such as clustered barrier loci and epistasis. It is also currently unclear how long heterogeneous species barriers may be detectable, as continued accumulation of barrier loci should eventually lead to genome-wide ‘coupling’ or ‘congealing’ [24,92], in which introgression is reduced globally rather than locally. Regardless of the answers to these questions, the recent evidence for highly polygenic species barriers highlights the dangers of assuming strictly neutral evolution when modelling speciation. Models that incorporate variable selection pressures among sites [69,93] are likely to be more realistic. Our results here are intriguing in that they show that, despite the widely distributed barriers across the genome, introgression has nonetheless dramatically reshaped species relationships. A few recent examples have shown how introgression can lead to different topologies across genome regions, but our data go further in showing how this phylogenetic heterogeneity can be predicted by recombination rate. Perhaps most intriguing is the fact that hybridisation between these butterflies is very rare, but has evidently been ongoing for long enough to broadly rewrite their evolutionary history. This raises questions about how we view even strongly isolated species as entities and the degree to which animal life can be accurately viewed as a bifurcating tree.

## Materials and methods

### Samples and genotyping

We used whole-genome resequencing data from 92 wild-caught butterflies (S1 Table) [1,49,52,65,81]. Reads were mapped to the *mel* genome assembly version 2 [62] using BWA mem version 0.7.2, using default parameters. Read depth was computed using GATK version 3.4 DepthOfCoverage [94]. Average read depth across all 92 samples was 29.22 (S1 Table). Genotyping was performed using GATK version 3.4 HaplotypeCaller and GenotypeGVCFs tools [94], using default parameters except that heterozygosity was set to 0.02. Each geographic population (10 samples each) was genotyped separately. Variant sites were accepted only if the quality (QUAL) value was ≥30, and individual genotype calls were accepted only where the sample depth of coverage for the position was ≥8. Scaffold positions in the Hmel2 assembly were converted to chromosome positions based on the most recent scaffolding [50], now released as Hmel2.5. Two sets of filtered SNPs were generated for the analyses below. In addition to the requirement of ≥8× depth of coverage, SNPs were required to be biallelic, and sites at which more than 75% of samples were heterozygous—or where the minor allele was present in only a single haploid copy—were discarded. SNP Set 1 had the further requirement that at least 9 out of the 10 samples representing each of the 9 ingroup populations, and 1 of the 2 outgroup samples, had an accepted genotype call, resulting in 14,406,386 SNPs. SNP Set 2 had the less stringent requirement that at least 4 of the samples from each ingroup population, and 1 of the outgroup samples, had an accepted genotype call, resulting in 23,084,596 SNPs.

### PCA

We used Eigenstrat SmartPCA [95] to investigate population structure and confirm sample identity. SNP Set 1 was used for this analysis, and the outgroup *num* samples were excluded.

### Phylogenetic network analysis

Pairwise absolute genetic distance between all pairs of samples was computed using the script distMat.py, available from github.com/simonhmartin/genomics_general. SNP Set 1 was used but with an added filter to retain only SNPs separated by at least 1 kb, to reduce computational time. A phylogenetic network was computed from the distance matrix using the NeighbourNet approach [96], implemented in SplitsTree version 4 [97], using default parameters.

### Topology weighting

To quantify genealogical relationships among taxa, we used topology weighting by iterative sampling of subtrees, *Twisst* [56] (github.com/simonhmartin/twisst). This also made use of SNP Set 1. Genotypes for all samples were first phased and imputed using SHAPEIT version 2 [98,99]. Neighbour-joining phylogenies were inferred for windows of 50 SNPs, following extensive simulations [56]. Using a fixed number of SNPs as opposed to a fixed absolute window size ensures sufficient information for reliable tree inference while minimising recombination within each analysed window [56]. Exact weightings were computed for all inferred genealogies that could be simplified to ≤2,000 remaining sample combinations (see reference [56] for details). In cases in which this was not possible, approximate weightings were computed by randomly sampling combinations of haplotypes until estimated weightings for all 15 possible topologies had a 95% binomial confidence interval of <0.05. Confidence intervals were computed according to the Wilson method, as implemented by the package binom [100] in R [101].

To guide our interpretation of the topology weighting results, we performed coalescent simulations using *msms* [102] under a simplified model that approximates the demographic history of these species. The main aims of these simulations were to determine (1) whether the level of phylogenetic discordance observed empirically could be explained purely as a result of ancestral ILS or whether it required the inference of introgression, and (2) whether a bias in the direction of gene flow produced skews in the weightings of certain topologies. We did not attempt to fit a complex model and infer the ‘true’ demographic history (i.e., split times, changes in *N*_*e*_, and the extent of gene flow over time) because this appears to be computationally intractable given current methods. Three different scenarios were considered, all with a split time of 6 million generations between the 2 pairs of ingroup taxa, corresponding to the approximately 1.5 million-year split between *cyd* and *mel* [45,57] (although this may be an overestimate [46]) and with constant population size (see S2 Fig for details). The first simulation had a normal population size of 2 million [52] and no gene flow. The second had an enlarged population size of 10 million and no gene flow. This size was selected post hoc to produce levels of phylogenetic discordance similar to those observed empirically. The third simulation had a population size of 2 million and included gene flow between 2 pairs of nonsister taxa. Gene flow was 4-fold greater in one direction than the other. To approximate a scenario in which some parts of the genome are resistant to gene flow, the migration rate was sampled from a gamma distribution with a shape parameter of 1 and scale of 3 (i.e., the distribution had an expected value of 3 migrants per generation). The scale (rate of migration) was selected post hoc to give a level of phylogenetic discordance similar to that observed empirically. For each scenario, we simulated 20,000 unlinked genealogies, with 10 representative tips for each of the 4 ingroup taxa and 1 for the outgroup, and computed topology weightings using *Twisst*, as described above.

### Admixture proportions

We estimated admixture proportions for 100 kb windows using *f*_d_ [18], which is based on the so-called ABBA-BABA test [103,104]. Unlike *Twisst*, this is a SNP-based method that does not assume a single genealogy per window but rather benefits from intrawindow recombination which increases the information content per window. The window size of 100 kb was selected because it provides sufficient resolution to capture variation while being large enough to minimise nonindependence among adjacent windows [52]. This analysis was implemented using the python script fourPopWindows.py, available from github.com/simonhmartin/genomics_general. To ensure that *f*_d_ is not affected by confounding factors such as *N*_e_ and selective sweeps, we first tested its performance in quantifying the proportion of admixture using coalescent simulations and compared it to other methods used to study admixture and genomic divergence (*F*_ST_, absolute genetic divergence [*d*_XY_], and Patterson’s *D* statistic) [103,104]. We used *msms* [102] to simulate the evolution of independent windows of 50 kb (100 windows for each population size and rate of gene flow), with a population recombination at rate of 1%. The models used were similar to those used for the topology weighting simulations above, except with 4 taxa rather than 5. They are shown, along with the range of *N*_e_ and rates of gene flow tested in S5 Fig.

Analyses of real data focused on quantifying admixture between the 2 sympatric species pairs: *cyd* and *mel*-W, and *tim* and *mel*-E. *f*_d_ was computed using a range of population combinations, including different allopatric control populations and either combining the 2 races that represent each broad geographic area (east of the Andes, west of the Andes, and French Guiana) or keeping them separate (S6 Fig). SNP Set 2 was used for these analyses, with the added requirement that, for the given run, at least 50% of samples in each population were genotyped and the outgroup was fixed for the ancestral state.

### Recombination rate estimation

Recombination rates were estimated in two different ways. First, we used the high-resolution linkage maps recently produced for *mel*, *cyd*, and hybrids [50] to estimate the local crossover rate. The 3 maps were produced from 335, 297, and 331 offspring, respectively. The recombination rate was computed as the slope of the locally weighted regression (loess span = 2 Mb) between physical position and map position along each chromosome [52,105]. Note that, because recombination is male limited in Lepidoptera, the values presented here represent the male-specific recombination rate. Conversion to an effective recombination rate at the population level would require knowledge of the effective sex ratio, which we do not have, so we chose here to use the male-specific rate. Second, we computed the population recombination rate for 100 kb windows using the maximum likelihood method implemented in LDHelmet [68]. This analysis was run separately for each population of 20 samples (i.e., combining races from the same area following the results of the PCA in Fig 1), using SNP Set 1, phased as described above. A window size of 50 SNPs was used, along with the recommended range of precomputed pairwise likelihoods. For convenience, ρ values were converted to cM/Mb by scaling values for each chromosome according to the map length of each chromosome, averaged across the 3 linkage maps used. As above, these values are therefore scaled to the male-specific recombination rate. The map-based approach has the advantage of providing an estimate of the true crossover recombination rate but suffers from limited resolution, whereas ρ can be estimated with high resolution but provides a composite of the crossover recombination rate and the *N*_*e*_, which can be biased by confounding factors such as selective sweeps that reduce *N*_*e*_ in some part of the genome. We therefore compared the two approaches to evaluate the extent to which the higher-resolution estimates or ρ provide reliable information about the underlying recombination rate.

### Data availability

Resequencing fastq files for all 92 individuals are available from the European Nucleotide Archive (accession numbers are provided in S1 Table). Filtered SNP data (VCF) and all processed data files used to generate all empirical and simulation figures are available from the Dryad digital repository: http://dx.doi.org/10.5061/dryad.sk2pd88 [55].

## Supporting information

S1 Fig Examples of topology weighting for 3 individual windows. Each tree (top), labelled X, Y, and Z, represents an inferred genealogy from a single 50 SNP window. Bar plots (middle) show the weightings for the 15 possible topologies for each tree. Tree X has a weighting of 1 for Topology 3, indicating complete monophyly of *cyd* with *tim* and *mel*-W with *mel*-E. Note that a tree can have a weighting of 1 for a single topology even if no individual taxon shows complete lineage sorting, as long as pairs of taxa are completely sorted (monophyletic). Tree Y is more complex, with four different topologies represented. However, the strongest weighting is for Topology 6. Tree Z is even more complex, and all 15 topologies are represented, indicating very little lineage sorting. At the bottom, the weightings are shown for a 250 Kb region of Chromosome 2, with the locations of windows X, Y, and Z indicated. Both stacked and overlapping plots are shown. *cyd*, *H*. *cydno*; *mel*, *H*. *melpomene*; *mel*-E, eastern races of *mel*; *mel*-W, western races of *mel*; *tim*, *H*. *timareta*.
(PNG)

S2 Fig Topology weightings for simulated genealogies. Coalescent simulations were performed using a simplified population history that approximately matches that for the studied species. Constant population size was used throughout, and split times are indicated. Three simulations were performed, one with normal population size (2 million) and no gene flow (top), one with a large population size (10 million) and no gene flow (middle), and one with a normal population size but with gene flow between two pairs of populations. Gene flow was uneven (four times stronger in one direction than the other, as indicated by arrows). To approximate a natural situation with some part of the genome resistant to introgression, the rate of gene flow was drawn from a gamma distribution with shape parameter 1 and scale 3 (i.e., an expected value of 3 migrants per generation). See Materials and methods for further details. For each scenario, average weightings across 20,000 simulated genealogies (solid bars) are shown for the 15 possible topologies (shown above). The percentage of genealogies with a maximal weighting (= 1) for each topology is also shown (shaded bars). The simulations show that without gene flow, we expect a considerably higher weighting for the species topology (T3), with less phylogenetic discordance and more lineage sorting than seen in the real data (Fig 2). Increasing the population size creates greater discordance but produces similar weightings for topologies T6 and T9. By contrast, adding gene flow also increases discordance but generates a bias towards far greater weighting of T6 than T9. The uneven gene flow also creates biases for T14 over T11 and T5 over T4. Therefore, the observed patterns in Fig 2 are more consistent with gene flow than with ancestral ILS. Data deposited in the Dryad repository [55]. ILS, incomplete lineage sorting.
(PNG)

S3 Fig Raw weightings for all topologies across all chromosomes. See Fig 2 for the colour legend. Raw values without smoothing are plotted here, unlike in Fig 3 of the main paper. Weightings are stacked so that all 15 topologies can be distinguished. Horizontal axis tick marks are spaced by 1 Mb. Note that the chromosomes are plotted in groups: first, the Z sex chromosome, then the 10 unfused (short) autosomes, and then the 10 fused (long) autosomes. Data deposited in the Dryad repository [55].
(PNG)

S4 Fig Trends in topology weightings among and within chromosomes. (A) The average weighting for all 15 topologies (colours as in Fig 2) for each of the 20 autosomes, plotted against the physical length of the chromosome. Fitted linear regressions are shown for reference. (B) Average weightings for all 15 topologies binned according to their relative chromosome position, from the centre (0) to the periphery (1). Each bin represents 5% of the chromosome arm, with the range indicated by a horizontal line. Vertical lines indicate ±1 SE. Data deposited in the Dryad repository [55].
(PNG)

S5 Fig Testing the robustness of *f*_d_ to estimate the admixture proportion. Simulations show that *f*_d_ is largely robust to *N*_e_. Sequences were simulated following the model on the left, with a range of different population sizes, indicated by different colours (2 are shown in the model for example). Simulated divergence times were chosen to approximate the splits between the outgroup silvaniform clade and the clade of *mel*, *cyd*, and *tim* (approximately 4 Mya [58]), and the divergence between *mel* and the ancestor of *cyd* and *tim* (1–1.5 Mya [45,46,57]). Population sizes ranging from 250,000 to 4,000,000 were tested. For comparison, other divergence and admixture statistics are included. Relative and absolute divergence statistics *F*_ST_ and *d*_XY_ are both strongly dependent on *N*_*e*_. Patterson’s D statistic is strongly affected by *N*_*e*_ and is nonlinear. By contrast, *f*_*d*_ is approximately proportional to the simulated level of migration and is largely unaffected by *N*_*e*_, except when *N*_*e*_ is large, in which case *f*_d_ tends to underestimate the simulated admixture proportion. This is consistent with a loss of power with reduced lineage sorting in large populations. *N*_*e*_ for *mel* was estimated to be 2–3 million [52], suggesting that admixture would indeed be weakly underestimated. However, as we are primarily interested in testing for reduced admixture in parts of the genome with reduced recombination rate, which usually corresponds to reduced *N*_*e*_ due to enhanced linked selection, the observed bias would have a conservative influence on our main analysis. We also tested simulated histories in which the donor and recipient populations undergo an expansion and contraction, respectively (second row), or the inverse (third row). Expansion of the donor population causes an exaggeration of the underestimate of admixture when *N*_*e*_ is large, but otherwise these changes do not have a significant effect on the performance of *f*_*d*_. All plotted data deposited in the Dryad repository [55]. *cyd*, *H*. *cydno*; *d*_XY_, absolute genetic divergence; *F*_ST_, fixation index; *mel*, *H*. *melpomene*; Mya, million years ago; *tim*, *H*. *timareta*.
(PNG)

S6 Fig Sets of taxa used to estimate admixture proportions using *f*_d_. Sets 1–3 were used to estimate admixture between *cyd* and *mel*-W. Sets 4–6 were used to estimate admixture between *tim* and *mel*-E. In each set, P2 and P3 represent the 2 sympatric populations between which the level of admixture is to be measured. P1 represents the allopatric ‘control’ population that is closely related to P2 but thought not to be subject to contemporary hybridisation with P3. The diagrams on the right show, for each set, the relationships among the 3 populations considered (bold lines), as well as the period over which admixture between P2 and P3 can be detected given P1 (shaded). In all sets, *num* was used as the outgroup. *ama*, *H*. *m*. *amaryllis*; *chi*, *H*. *c*. *chioneus*; *cyd*, *H*. *cydno*; *flo*, *H*. *t*. *Florencia*; *mal*, *H*. *m*. *malleti*; *mel*-E, eastern races of *mel*; *mel*-G, *H*. *m*. *melpomene* from French Guiana; *mel*-W, western races of *mel*; *ros*, *H*. *m*. *rosina*; *tim*, *H*. *timareta*; *txn*, *H*. *t*. *Thelxinoe*; *vul*, *H*. *m*. *vulcanus*; *zel*, *H*. *c*. *zelinde*.
(PNG)

S7 Fig Fine-scale patterns of admixture between *cyd* and *mel*-W. Estimated admixture proportion (*f*_d_) computed in 20 Kb sliding windows, sliding in increments of 5 Kb. Note that the chromosomes are plotted in groups: first, the Z sex chromosome, then the 10 unfused (short) autosomes, and then the 10 fused (long) autosomes. Red boxes indicate the locations of 3 known wing-patterning genes (+/−100 kb): *wnt-A* (Chromosome 10), *cortex* (Chromosome 15), and *optix* (Chromosome 18). *f*_d_ was computed between *cyd* and *mel*-W using Set 1a (solid line) and Set 1b (dashed) (see S6 Fig). In nearly all cases, there is reduced admixture between *cyd* and *mel*-W in the vicinity of the wing-patterning genes, consistent with localised barriers to introgression. The one exception is the cortex locus on Chromosome 15, at which there is elevated admixture for Set 1b (i.e., between *H*. *cydno zelinde* and *H*. *melpomene vulcanus*). This has in fact been previously recorded as a probable rare instance of introgression of a wing-patterning allele between *cyd* and *mel* [49]. This allele appears to be responsible for the dorsal melanisation of the hindwing yellow bar in *H*. *m*. *vulcanus*. Therefore, these loci provide robust support for the use of *f*_d_ to quantify admixture between these taxa. Black boxes indicate the fusion points of the fused chromosomes [62] (+/−100 kb). There is no consistent trend of reduced admixture around the fusion points. Data deposited in the Dryad repository [55]. cyd, *H*. *cydno*; *mel*, *H*. *melpomene*; *mel*-W, western races of *mel*.
(PNG)

S8 Fig Fine-scale patterns of admixture between *tim* and *mel*-E. Estimated admixture proportion (*f*_d_) computed in 20 Kb sliding windows, sliding in increments of 5 Kb. Note that the chromosomes are plotted in groups: first, the Z sex chromosome, then the 10 unfused (short) autosomes, and then the 10 fused (long) autosomes. Three known wing-patterning genes are indicated by red boxes: *wnt-A* (Chromosome 10), *cortex* (Chromosome 15), and *optix* (Chromosome 18). *f*_d_ was computed between *tim* and *mel*-E using Set 4a (solid line) and Set 4b (dashed) (see S6 Fig). In all cases, there is elevated admixture between *tim* and *mel*-E in the vicinity of the wing-patterning genes, consistent with localised introgression of wing-patterning alleles. Black boxes indicate the fusion points of the fused chromosomes [62] (+/−100 kb). There is no consistent trend of reduced admixture around the fusion points. Data deposited in the Dryad repository [55]. *mel*, *H*. *melpomene*; *mel*-E, eastern races of *mel*; *tim*, *H*. *timareta*.
(PNG)

S9 Fig Recombination rates plotted across chromosomes. Solid lines show the crossover recombination rate estimated from linkage maps [50]. Dashed lines show the maximum likelihood estimate for ρ, computed for 100 Kb windows separately for *cyd*, *tim*, *mel*-W, and *mel*-E (indicated by colours). The black dashed line indicates the mean ρ across the four populations, plotted as a locally weighted average (loess span = 2 Mb). Data deposited in the Dryad repository [55]. ρ, population recombination rate; *cyd*, *H*. *cydno*; *mel*, *H*. *melpomene*; *mel*-E, eastern races of *mel*; *mel*-W, western races of *mel*; *tim*, *H*. *timareta*.
(PNG)

S10 Fig Relationship between recombination rate and gene density. Gene density (i.e., the proportion of coding sequence) in 100 kb windows plotted against ρ (left) and crossover recombination rate (right). The line shows a fitted linear regression. While there is a strong negative relationship between ρ and gene density, this may partly reflect the fact that ρ represents a composite of recombination and local *N*_e_, which will tend to be lower in regions of high gene density due to linked selection [52]. Nevertheless, there is also a negative relationship between the crossover recombination rate and gene density, indicating that regions of lower recombination do indeed tend to harbour more coding sequence. The relationship is fairly weak, but it is unclear to what extent this might reflect the inaccuracies of measuring local recombination rates based on linkage mapping [50]. Data deposited in the Dryad repository [55]. ρ, population recombination rate; *N*_e_, effective population size.
(PNG)

S11 Fig Admixture is positively correlated with recombination rate. Admixture proportions estimated for nonoverlapping 100 kb windows, plotted against the local recombination rate, computed as ρ rescaled to cM/Mb (panel A) or estimated directly from linkage maps (panel B). Solid lines indicate the locally weighted average (loess span = 0.75). Admixture between *cyd* and *mel*-W (Sets 1–3, see S6 Fig) as well as that between *tim* and *mel*-E (Sets 4–6) increases nonlinearly with increasing recombination rate, with the exception of Set 6, for which admixture proportions are low, and there is only evidence for a weak positive relationship in windows of low recombination rate. This may be driven by the close relationship and likely ongoing migration between *mel*-E and *mel*-G (see Figs 1 and S6), which could limit our ability to detect admixture between *tim* and *mel*-E. The estimated admixture proportion between *cyd* and *mel*-W using Set 3 is also much lower than for Sets 1 and 2. This may be driven by strongly directional introgression from *cyd* into *mel*-W, which is also indicated by topology weightings, as described in the main paper. If introgression is largely in the direction from P2 into P3, *f*_d_ tends to underestimate the true admixture proportion [18]. Data deposited in the Dryad repository [55]. ρ, population recombination rate; *cyd*, *H*. *cydno*; *f*_d_,; *mel*, *H*. *melpomene*; *mel*-E, eastern races of *mel*; *mel*-G, French Guiana *mel*; *mel*-W, western races of *mel*; *tim*, *H*. *timareta*.
(PNG)

S12 Fig Admixture is negatively correlated with the proportion of coding sequence. Admixture proportions estimated for nonoverlapping 100 Kb windows, plotted against the proportion of coding sequence. Solid lines indicate the locally weighted average (loess span = 0.75). Explanations for the lower average levels of admixture in Sets 3 and 6 are discussed in the legend of S11 Fig above. Data deposited in the Dryad repository [55].
(PNG)

S13 Fig Within-chromosome correlations between admixture and recombination rate. Locally smoothed regressions (loess, span = 1) of estimated admixture proportions between *cyd* and *mel*-W (Set 1) (panel A) and between *tim* and *mel*-E (Set 4) (panel B) for 100 kb windows, against ρ. Individual chromosomes are plotted separately. Asterisks at the end of each line indicate a positive Spearman rank correlation (*p* ≤ 0.05) for that chromosome. Solid lines represent the longer fused autosomes, and dashed lines indicate the shorter unfused autosomes. The fine dashed line indicates the Z sex chromosome. Lines along the top indicate recombination rate bins for which comparisons were made between the 2 chromosome types. Asterisks along the top indicate a significantly lower admixture proportion on average in fused than unfused autosomes for the given bin (Mann-Whitney U test, *p* ≤ 0.05). Comparisons were made up to 7 cM/Mb, because beyond this, there were too few regions on fused chromosomes for reliable comparison. Data deposited in the Dryad repository [55]. ρ, population recombination rate; *cyd*, *H*. *cydno*; *mel*, *H*. *melpomene*; *mel*-E, eastern races of *mel*; *mel*-W, western races of *mel*; *tim*, *H*. *timareta*.
(PNG)

S1 Table Sample and sequencing information. (PDF)

S2 Table Correlations between admixture proportion (*f*_d_) and recombination rate. (PDF)

## Abbreviations

ρ: population recombination rate
cyd: *H*. *cydno*
DMI: Dobzhansky-Muller incompatibility
*F*_ST_: fixation index
ILS: incomplete lineage sorting
LD: linkage disequilibrium
mel: *H*. *melpomene*
*mel*-E: eastern races of *mel*
*mel*-G: French Guiana *mel*
*mel-*W: western races of *mel*
*N*_e_: effective population size
num: *H*. *numata*
PCA: principal components analysis
QTL: quantitative trait locus
r: per-generation recombination rate
SNP: single-nucleotide polymorphism
tim: *H*. *timareta*
