# Supplementary figures and images for: Recombination rate variation shapes barriers to introgression across butterfly genomes
Source: PLoS Biol. 2019 Feb 7;17(2):e2006288. doi: 10.1371/journal.pbio.2006288 (PMC6366726; doi:10.1371/journal.pbio.2006288)

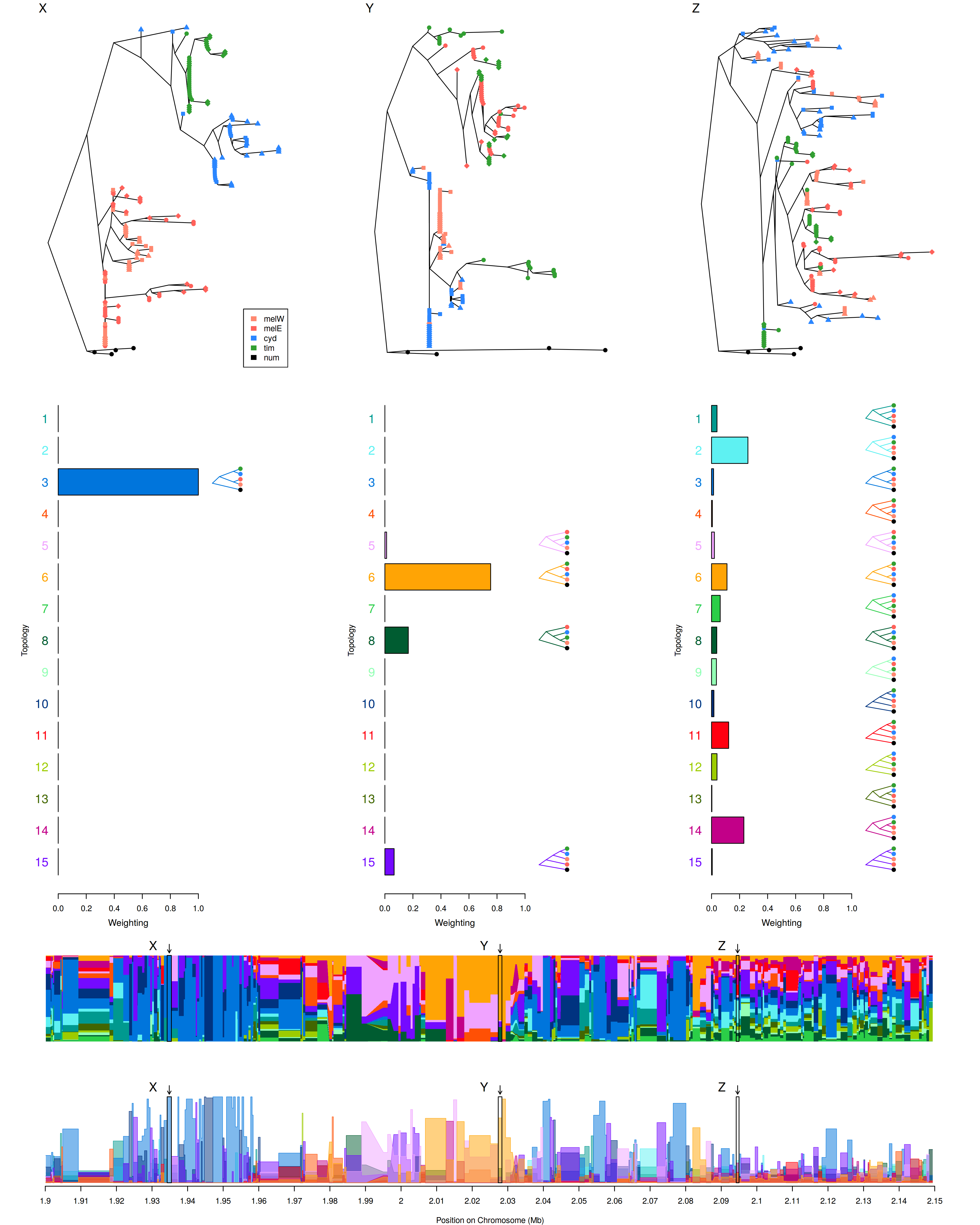

Supplement: S1 Fig — Each tree (top), labelled X, Y, and Z, represents an inferred genealogy from a single 50 SNP window. Bar plots (middle) show the weightings for the 15 possible topologies for each tree. Tree X has a weighting of 1 for Topology 3, indicating complete monophyly of cyd with tim and mel-W with mel-E. Note that a tree can have a weighting of 1 for a single topology even if no individual taxon shows complete lineage sorting, as long as pairs of taxa are completely sorted (monophyletic). Tree Y is more complex, with four different topologies represented. However, the strongest weighting is for Topology 6. Tree Z is even more complex, and all 15 topologies are represented, indicating very little lineage sorting. At the bottom, the weightings are shown for a 250 Kb region of Chromosome 2, with the locations of windows X, Y, and Z indicated. Both stacked and overlapping plots are shown. cyd, H. cydno; mel, H. melpomene; mel-E, eastern races of mel; mel-W, western races of mel; tim, H. timareta. (PNG) [file pbio.2006288.s001.png]

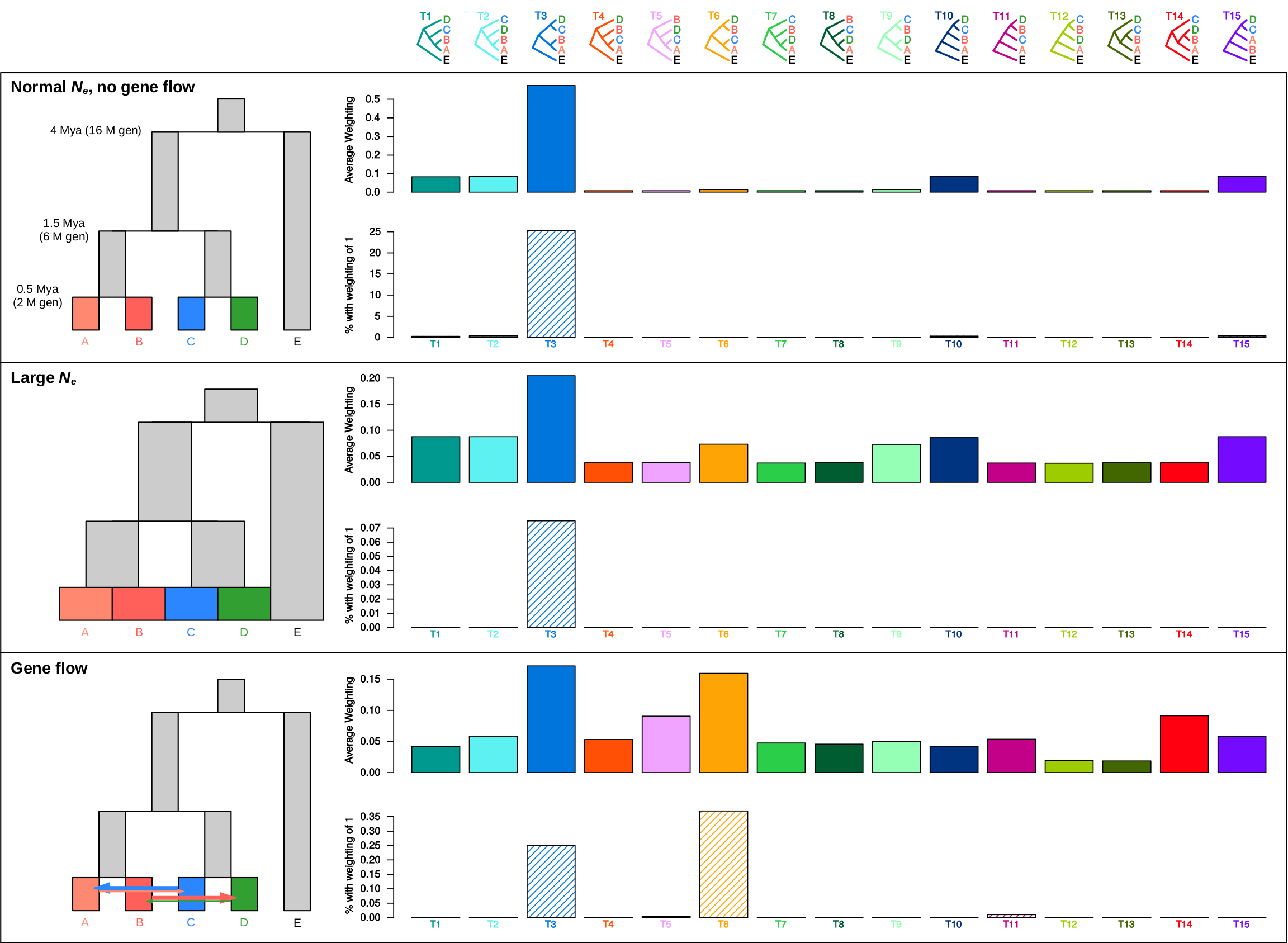

Supplement: S2 Fig — Coalescent simulations were performed using a simplified population history that approximately matches that for the studied species. Constant population size was used throughout, and split times are indicated. Three simulations were performed, one with normal population size (2 million) and no gene flow (top), one with a large population size (10 million) and no gene flow (middle), and one with a normal population size but with gene flow between two pairs of populations. Gene flow was uneven (four times stronger in one direction than the other, as indicated by arrows). To approximate a natural situation with some part of the genome resistant to introgression, the rate of gene flow was drawn from a gamma distribution with shape parameter 1 and scale 3 (i.e., an expected value of 3 migrants per generation). See Materials and methods for further details. For each scenario, average weightings across 20,000 simulated genealogies (solid bars) are shown for the 15 possible topologies (shown above). The percentage of genealogies with a maximal weighting (= 1) for each topology is also shown (shaded bars). The simulations show that without gene flow, we expect a considerably higher weighting for the species topology (T3), with less phylogenetic discordance and more lineage sorting than seen in the real data (Fig 2). Increasing the population size creates greater discordance but produces similar weightings for topologies T6 and T9. By contrast, adding gene flow also increases discordance but generates a bias towards far greater weighting of T6 than T9. The uneven gene flow also creates biases for T14 over T11 and T5 over T4. Therefore, the observed patterns in Fig 2 are more consistent with gene flow than with ancestral ILS. Data deposited in the Dryad repository [55]. ILS, incomplete lineage sorting. (PNG) [file pbio.2006288.s002.png]

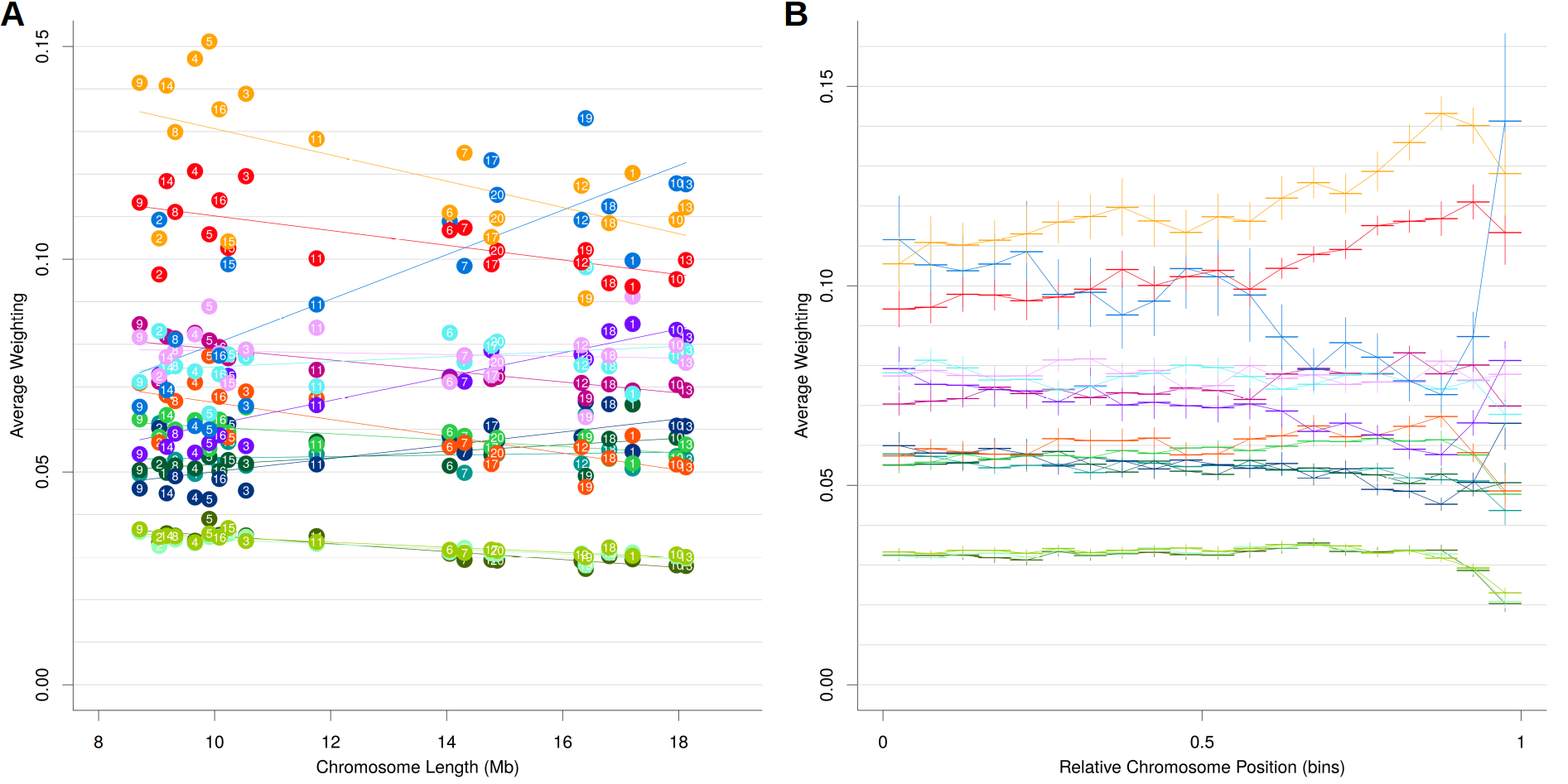

Supplement: S4 Fig — (A) The average weighting for all 15 topologies (colours as in Fig 2) for each of the 20 autosomes, plotted against the physical length of the chromosome. Fitted linear regressions are shown for reference. (B) Average weightings for all 15 topologies binned according to their relative chromosome position, from the centre (0) to the periphery (1). Each bin represents 5% of the chromosome arm, with the range indicated by a horizontal line. Vertical lines indicate ±1 SE. Data deposited in the Dryad repository [55]. (PNG) [file pbio.2006288.s004.png]

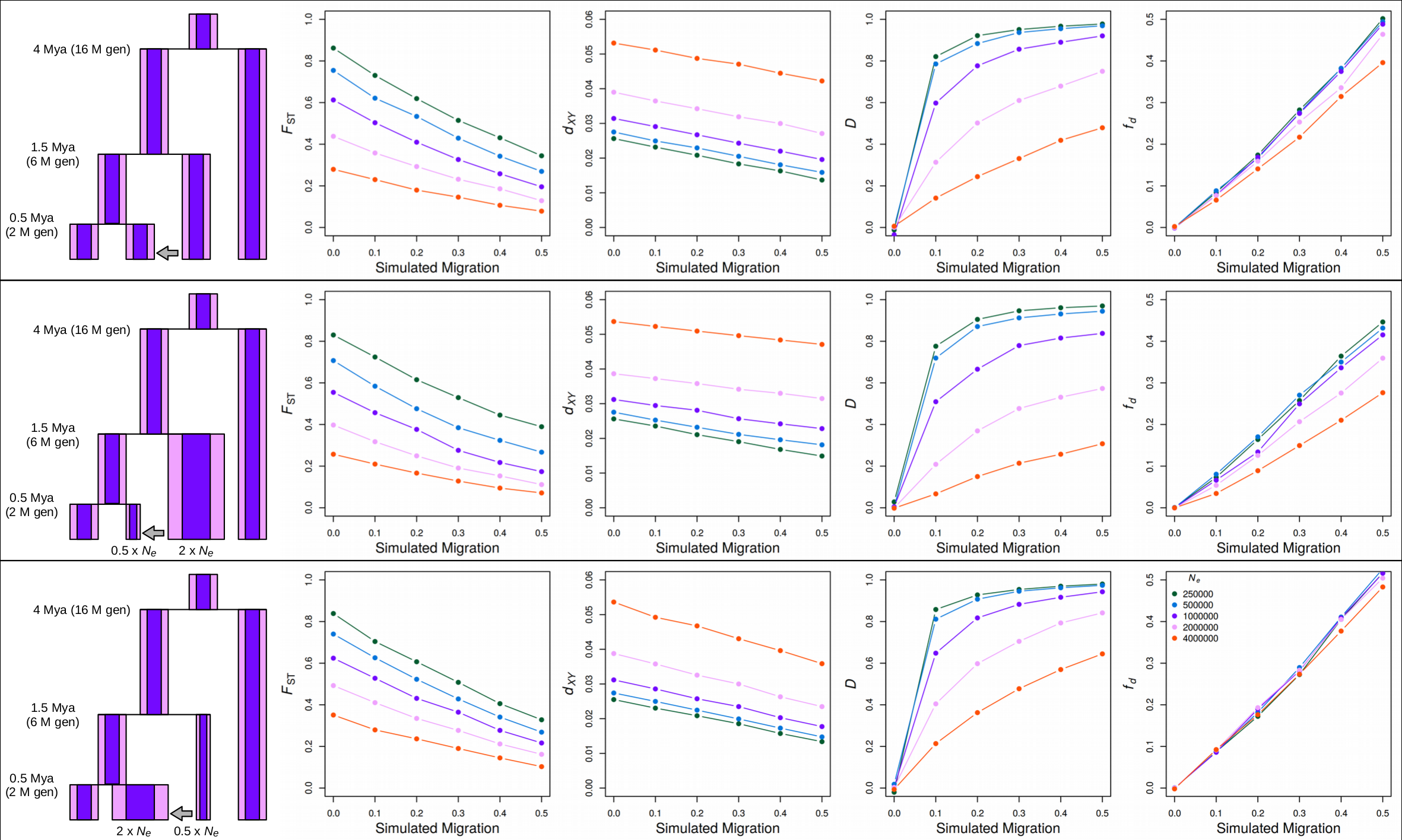

Supplement: S5 Fig — Simulations show that fd is largely robust to Ne. Sequences were simulated following the model on the left, with a range of different population sizes, indicated by different colours (2 are shown in the model for example). Simulated divergence times were chosen to approximate the splits between the outgroup silvaniform clade and the clade of mel, cyd, and tim (approximately 4 Mya [58]), and the divergence between mel and the ancestor of cyd and tim (1–1.5 Mya [45,46,57]). Population sizes ranging from 250,000 to 4,000,000 were tested. For comparison, other divergence and admixture statistics are included. Relative and absolute divergence statistics FST and dXY are both strongly dependent on Ne. Patterson’s D statistic is strongly affected by Ne and is nonlinear. By contrast, fd is approximately proportional to the simulated level of migration and is largely unaffected by Ne, except when Ne is large, in which case fd tends to underestimate the simulated admixture proportion. This is consistent with a loss of power with reduced lineage sorting in large populations. Ne for mel was estimated to be 2–3 million [52], suggesting that admixture would indeed be weakly underestimated. However, as we are primarily interested in testing for reduced admixture in parts of the genome with reduced recombination rate, which usually corresponds to reduced Ne due to enhanced linked selection, the observed bias would have a conservative influence on our main analysis. We also tested simulated histories in which the donor and recipient populations undergo an expansion and contraction, respectively (second row), or the inverse (third row). Expansion of the donor population causes an exaggeration of the underestimate of admixture when Ne is large, but otherwise these changes do not have a significant effect on the performance of fd. All plotted data deposited in the Dryad repository [55]. cyd, H. cydno; dXY, absolute genetic divergence; FST, fixation index; mel, H. melpomene; Mya, million [file pbio.2006288.s005.png]

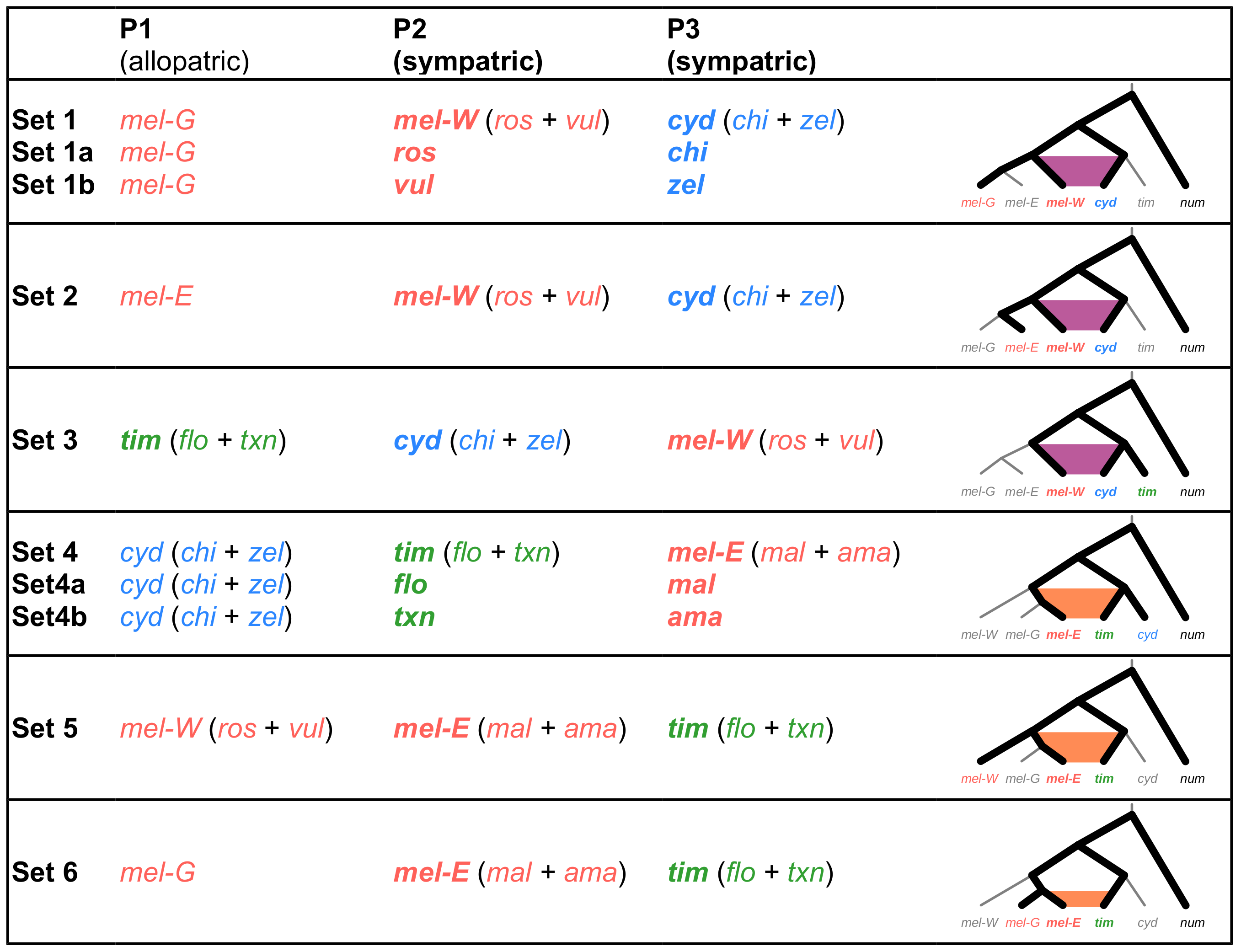

Supplement: S6 Fig — Sets 1–3 were used to estimate admixture between cyd and mel-W. Sets 4–6 were used to estimate admixture between tim and mel-E. In each set, P2 and P3 represent the 2 sympatric populations between which the level of admixture is to be measured. P1 represents the allopatric ‘control’ population that is closely related to P2 but thought not to be subject to contemporary hybridisation with P3. The diagrams on the right show, for each set, the relationships among the 3 populations considered (bold lines), as well as the period over which admixture between P2 and P3 can be detected given P1 (shaded). In all sets, num was used as the outgroup. ama, H. m. amaryllis; chi, H. c. chioneus; cyd, H. cydno; flo, H. t. Florencia; mal, H. m. malleti; mel-E, eastern races of mel; mel-G, H. m. melpomene from French Guiana; mel-W, western races of mel; ros, H. m. rosina; tim, H. timareta; txn, H. t. Thelxinoe; vul, H. m. vulcanus; zel, H. c. zelinde. (PNG) [file pbio.2006288.s006.png]

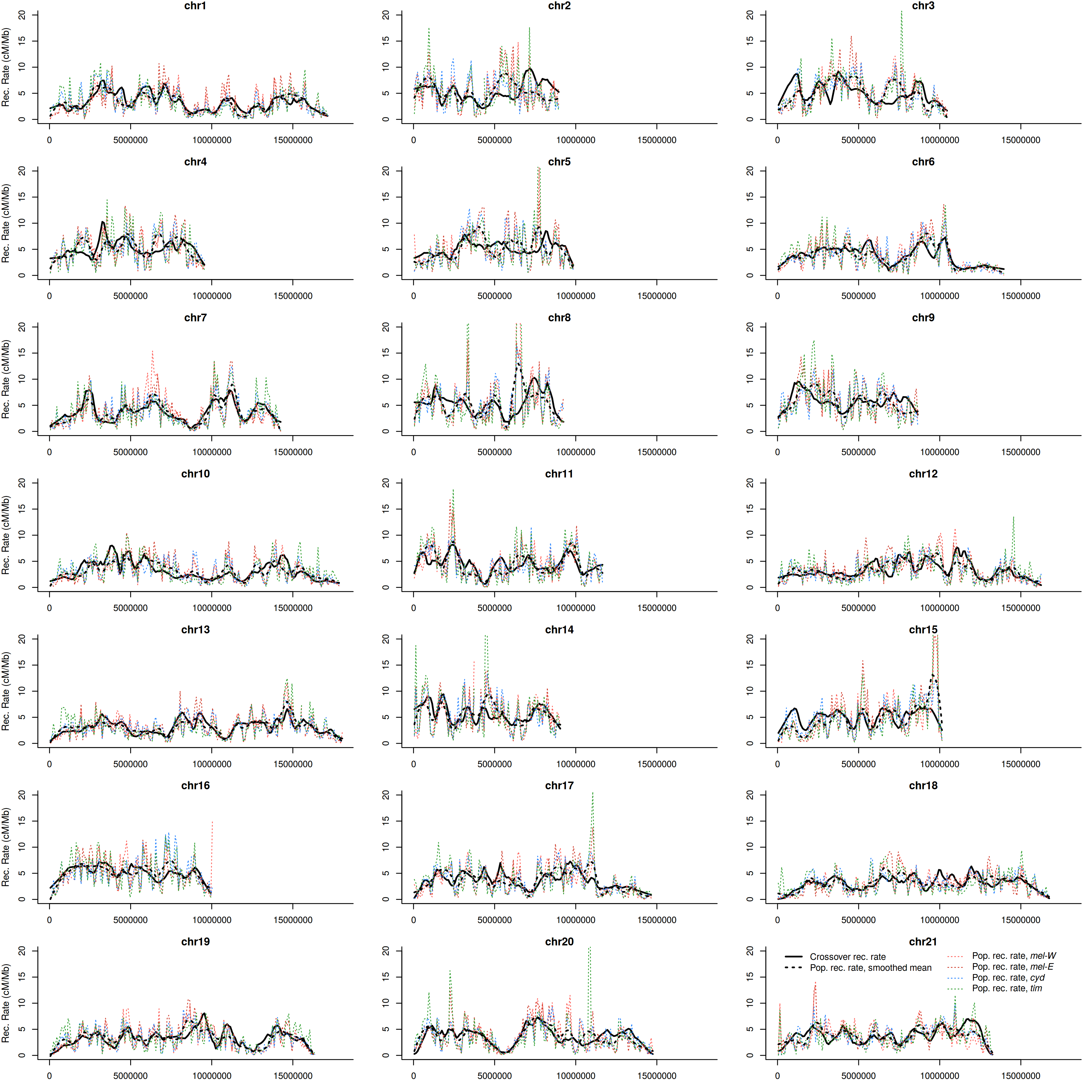

Supplement: S9 Fig — Solid lines show the crossover recombination rate estimated from linkage maps [50]. Dashed lines show the maximum likelihood estimate for ρ, computed for 100 Kb windows separately for cyd, tim, mel-W, and mel-E (indicated by colours). The black dashed line indicates the mean ρ across the four populations, plotted as a locally weighted average (loess span = 2 Mb). Data deposited in the Dryad repository [55]. ρ, population recombination rate; cyd, H. cydno; mel, H. melpomene; mel-E, eastern races of mel; mel-W, western races of mel; tim, H. timareta. (PNG) [file pbio.2006288.s009.png]

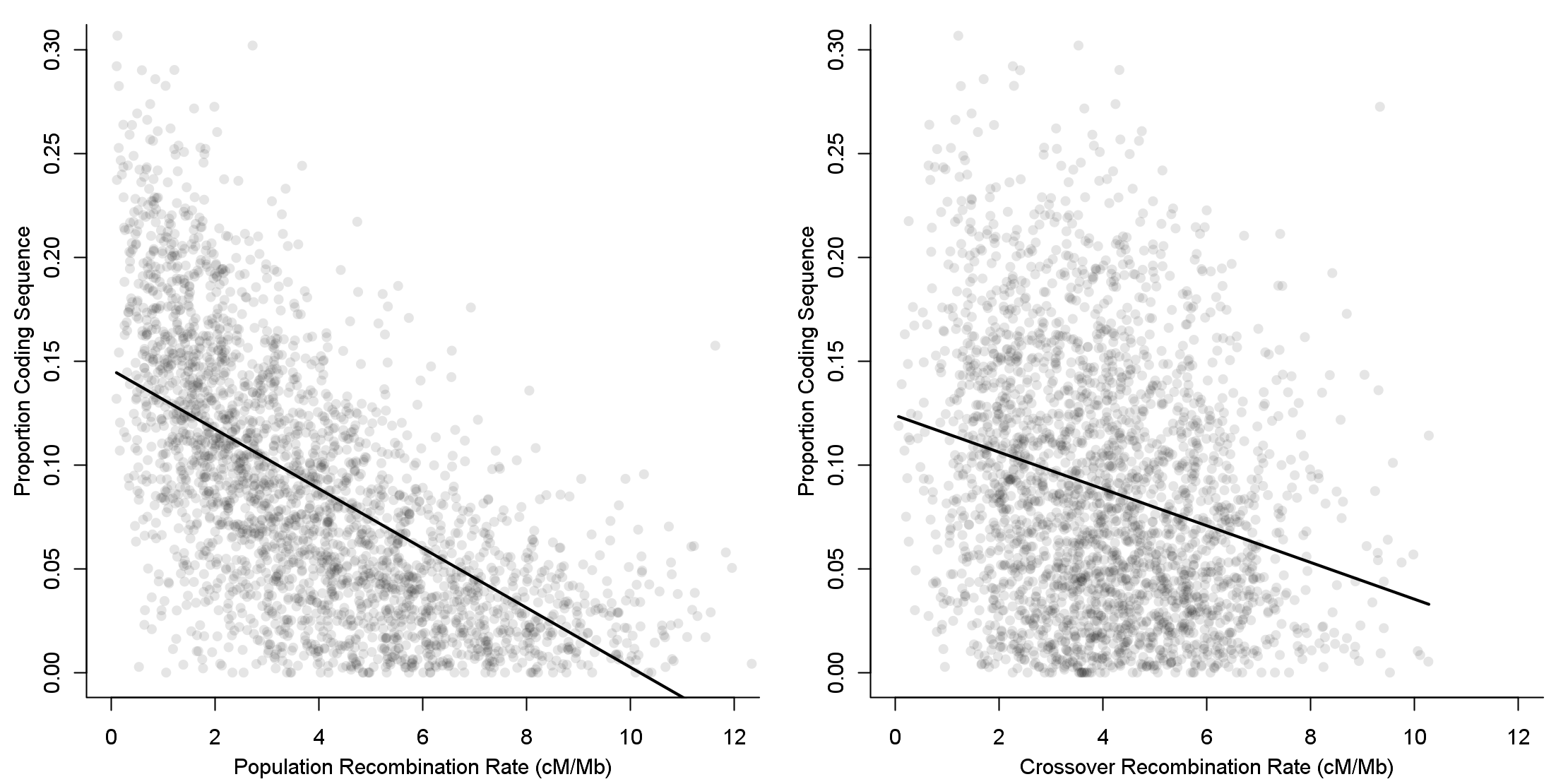

Supplement: S10 Fig — Gene density (i.e., the proportion of coding sequence) in 100 kb windows plotted against ρ (left) and crossover recombination rate (right). The line shows a fitted linear regression. While there is a strong negative relationship between ρ and gene density, this may partly reflect the fact that ρ represents a composite of recombination and local Ne, which will tend to be lower in regions of high gene density due to linked selection [52]. Nevertheless, there is also a negative relationship between the crossover recombination rate and gene density, indicating that regions of lower recombination do indeed tend to harbour more coding sequence. The relationship is fairly weak, but it is unclear to what extent this might reflect the inaccuracies of measuring local recombination rates based on linkage mapping [50]. Data deposited in the Dryad repository [55]. ρ, population recombination rate; Ne, effective population size. (PNG) [file pbio.2006288.s010.png]

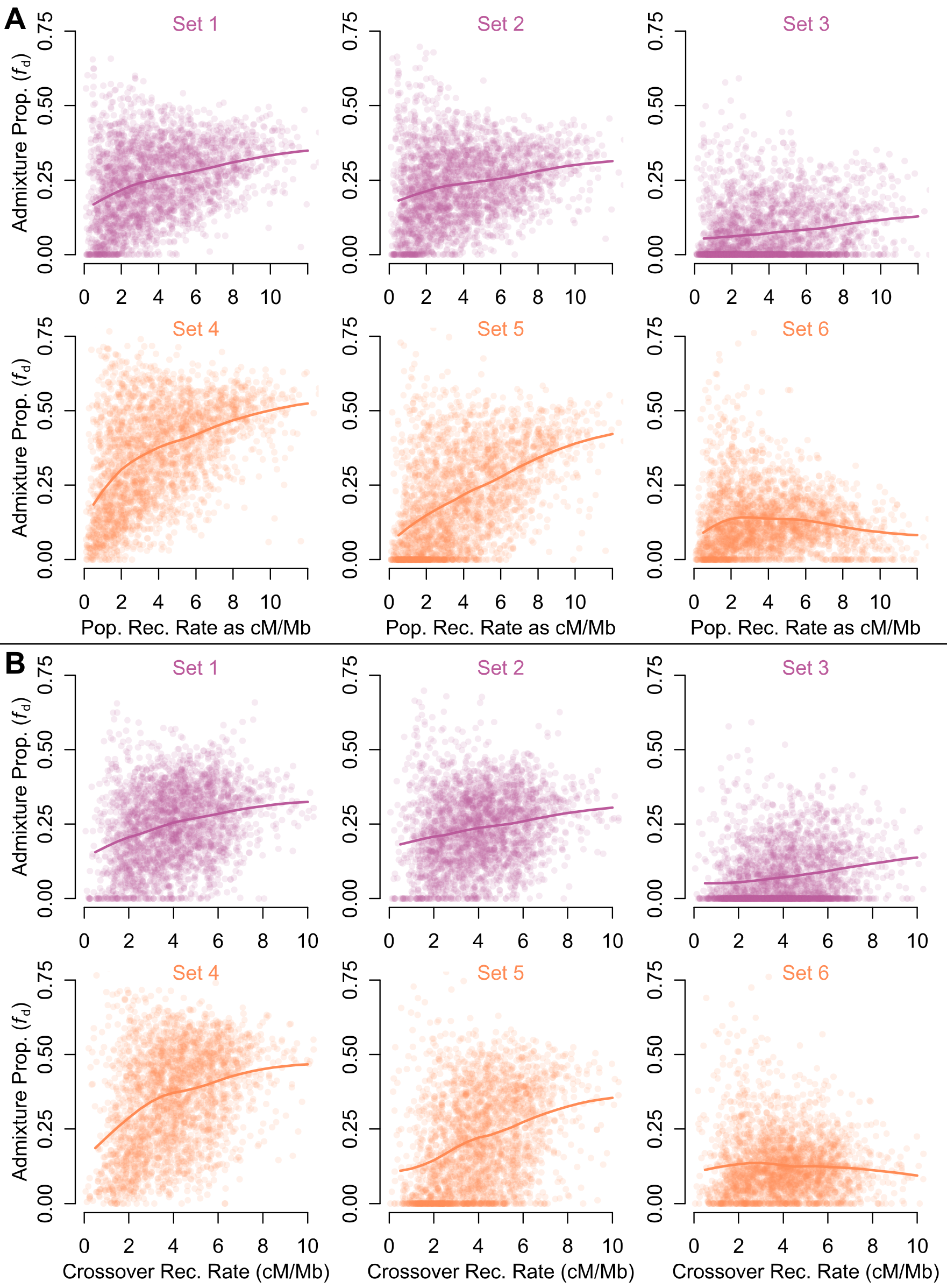

Supplement: S11 Fig — Admixture proportions estimated for nonoverlapping 100 kb windows, plotted against the local recombination rate, computed as ρ rescaled to cM/Mb (panel A) or estimated directly from linkage maps (panel B). Solid lines indicate the locally weighted average (loess span = 0.75). Admixture between cyd and mel-W (Sets 1–3, see S6 Fig) as well as that between tim and mel-E (Sets 4–6) increases nonlinearly with increasing recombination rate, with the exception of Set 6, for which admixture proportions are low, and there is only evidence for a weak positive relationship in windows of low recombination rate. This may be driven by the close relationship and likely ongoing migration between mel-E and mel-G (see Figs 1 and S6), which could limit our ability to detect admixture between tim and mel-E. The estimated admixture proportion between cyd and mel-W using Set 3 is also much lower than for Sets 1 and 2. This may be driven by strongly directional introgression from cyd into mel-W, which is also indicated by topology weightings, as described in the main paper. If introgression is largely in the direction from P2 into P3, fd tends to underestimate the true admixture proportion [18]. Data deposited in the Dryad repository [55]. ρ, population recombination rate; cyd, H. cydno; fd,; mel, H. melpomene; mel-E, eastern races of mel; mel-G, French Guiana mel; mel-W, western races of mel; tim, H. timareta. (PNG) [file pbio.2006288.s011.png]

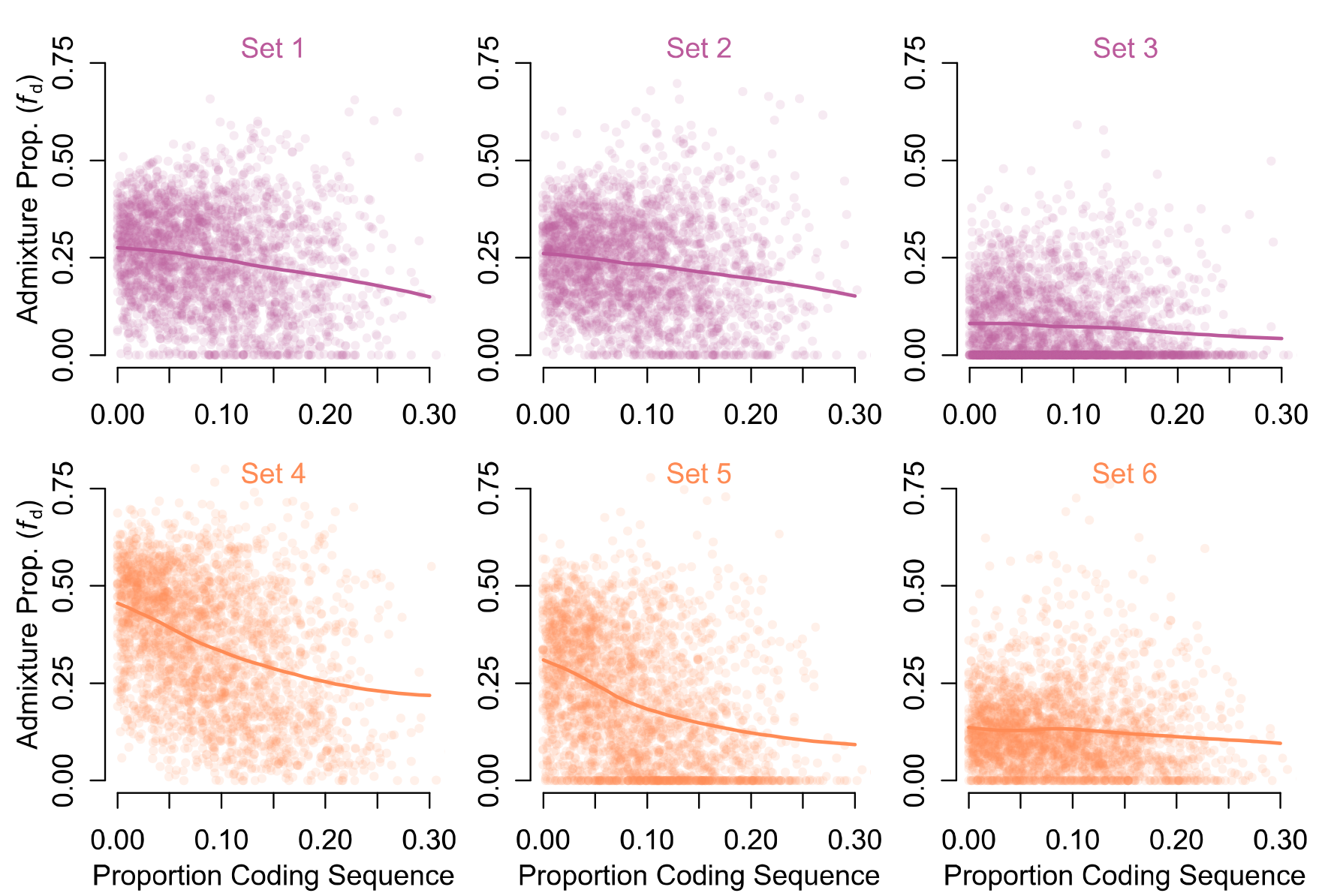

Supplement: S12 Fig — Admixture proportions estimated for nonoverlapping 100 Kb windows, plotted against the proportion of coding sequence. Solid lines indicate the locally weighted average (loess span = 0.75). Explanations for the lower average levels of admixture in Sets 3 and 6 are discussed in the legend of S11 Fig above. Data deposited in the Dryad repository [55]. (PNG) [file pbio.2006288.s012.png]

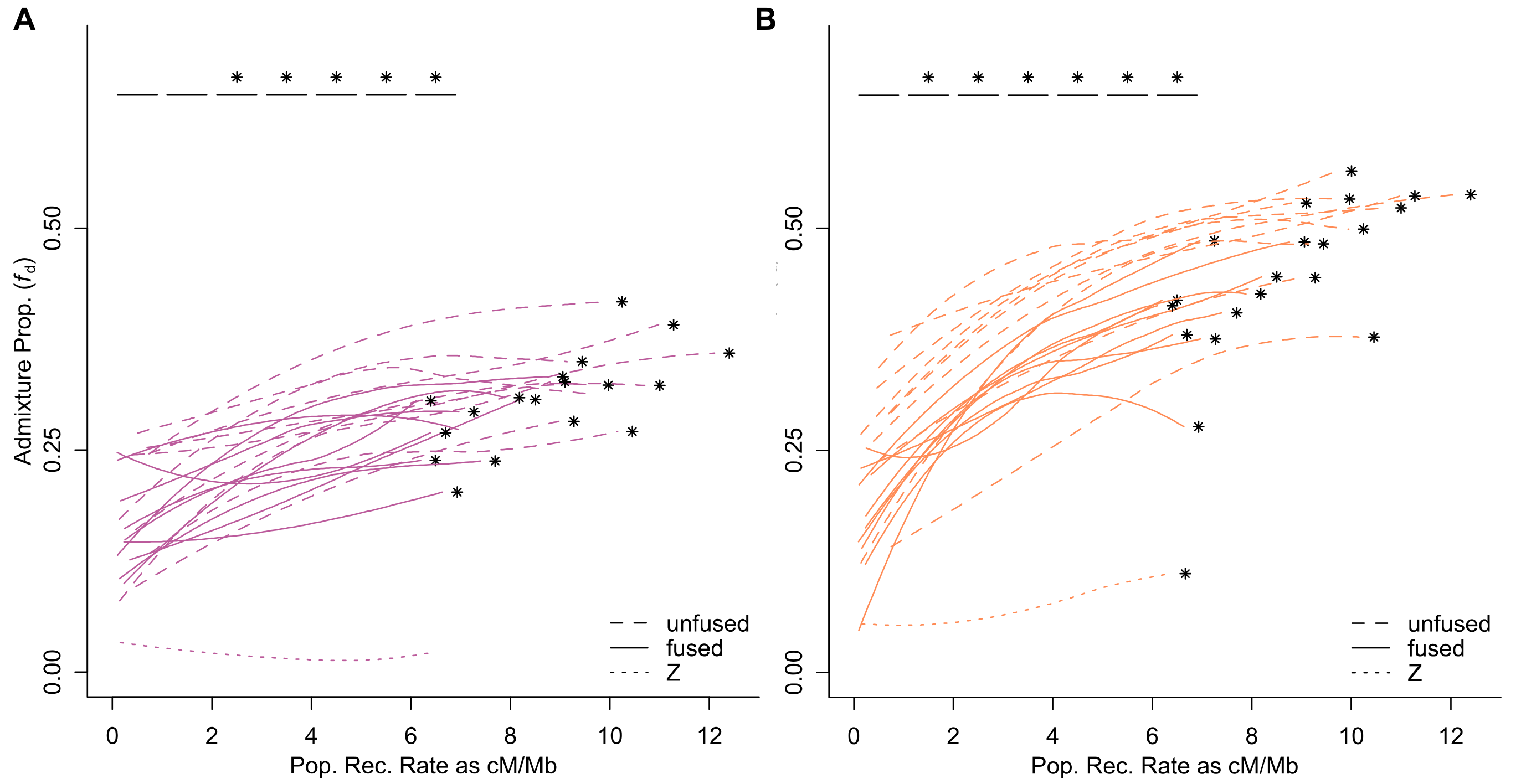

Supplement: S13 Fig — Locally smoothed regressions (loess, span = 1) of estimated admixture proportions between cyd and mel-W (Set 1) (panel A) and between tim and mel-E (Set 4) (panel B) for 100 kb windows, against ρ. Individual chromosomes are plotted separately. Asterisks at the end of each line indicate a positive Spearman rank correlation (p ≤ 0.05) for that chromosome. Solid lines represent the longer fused autosomes, and dashed lines indicate the shorter unfused autosomes. The fine dashed line indicates the Z sex chromosome. Lines along the top indicate recombination rate bins for which comparisons were made between the 2 chromosome types. Asterisks along the top indicate a significantly lower admixture proportion on average in fused than unfused autosomes for the given bin (Mann-Whitney U test, p ≤ 0.05). Comparisons were made up to 7 cM/Mb, because beyond this, there were too few regions on fused chromosomes for reliable comparison. Data deposited in the Dryad repository [55]. ρ, population recombination rate; cyd, H. cydno; mel, H. melpomene; mel-E, eastern races of mel; mel-W, western races of mel; tim, H. timareta. (PNG) [file pbio.2006288.s013.png]
