# Supplementary material for: Recombination rate variation shapes barriers to introgression across butterfly genomes
Source: PLoS Biol. 2019 Feb 7;17(2):e2006288. doi: 10.1371/journal.pbio.2006288 (PMC6366726; doi:10.1371/journal.pbio.2006288)
Supplement: S1 Table — (PDF) [file pbio.2006288.s014.pdf]

S1 Table. Sample and sequencing information

sample ID	Sequence ID	Taxon	Country	Sex	Longitude	Latitude	Accession (ENA, NCBI)	Ref	Total Gbp	Mean depth
CAM025091	chi.CAM25091	*H. cydno chioneus*	Panama	f	9.1200	-79.7020	SAMEA104585050	[81]	7.05	25.71
CAM025137	chi.CAM25137	*H. cydno chioneus*	Panama	f	9.1200	-79.7020	SAMEA104585051	[81]	7.54	27.5
CAM000580	chi.CAM580	*H. cydno chioneus*	Panama	m	9.1200	-79.7020	SAMEA104585044	[81]	6.16	22.45
CAM000582	chi.CAM582	*H. cydno chioneus*	Panama	m	9.1200	-79.7020	SAMEA104585045	[81]	6.68	24.36
CAM000585	chi.CAM585	*H. cydno chioneus*	Panama	m	9.1200	-79.7020	SAMEA104585047	[81]	6.30	22.98
CAM000586	chi.CAM586	*H. cydno chioneus*	Panama	m	9.1200	-79.7020	SAMEA104585048	[81]	6.37	23.24
CAM000553	chi.CJ553	*H. cydno chioneus*	Panama	m	9.1714	-79.7573	SAMEA1919256	[1]	9.69	35.33
CAM000560	chi.CJ560	*H. cydno chioneus*	Panama	m	9.1714	-79.7573	SAMEA1919265	[1]	9.55	34.84
CAM000564	chi.CJ564	*H. cydno chioneus*	Panama	m	9.1714	-79.7573	SAMEA1919278	[1]	10.63	38.75
CAM000565	chi.CJ565	*H. cydno chioneus*	Panama	m	9.1714	-79.7573	SAMEA1919262	[1]	12.50	45.58
CS002242	zel.CS1	*H. cydno zelinde*	Colombia	m	3.9394	-77.3689	SAMEA104106540	[49]	9.88	36.04
CS001028	zel.CS1028	*H. cydno zelinde*	Colombia	m	3.9583	-77.3733	SAMEA104585054	[81]	8.94	32.59
CS001029	zel.CS1029	*H. cydno zelinde*	Colombia	m	3.9394	-77.3689	SAMEA104585055	[81]	6.70	24.44
CS001030	zel.CS1030	*H. cydno zelinde*	Colombia	m	3.9394	-77.3689	SAMEA104585056	[81]	7.35	26.8
CS001033	zel.CS1033	*H. cydno zelinde*	Colombia	m	3.9583	-77.3733	SAMEA104585057	[81]	7.73	28.18
CS001035	zel.CS1035	*H. cydno zelinde*	Colombia	m	3.9583	-77.3733	SAMEA104585058	[81]	8.32	30.34
CS002261	zel.CS2	*H. cydno zelinde*	Colombia	m	3.9394	-77.3689	SAMEA104106542	[49]	9.10	33.2
CS002262	zel.CS2262	*H. cydno zelinde*	Colombia	f	3.9583	-77.3733	SAMEA3670517	[49]	4.91	17.91
CS000273	zel.CS273	*H. cydno zelinde*	Colombia	m	3.9583	-77.3733	SAMEA104585059	[81]	7.11	25.93
CS002260	zel.CS30	*H. cydno zelinde*	Colombia	f	3.9394	-77.3689	SAMEA104106543	[49]	11.35	41.38
JM-09-313	thxn.JM313	*H. timareta thelxinoe*	Peru	m	-6.4584	-76.2877	SAMEA1919266	[1]	9.84	35.89
JM-09-57	thxn.JM57	*H. timareta thelxinoe*	Peru	m	-6.4528	-76.2987	SAMEA1919254	[1]	12.52	45.64
JM-09-84	thxn.JM84	*H. timareta thelxinoe*	Peru	m	-6.4528	-76.2987	SAMEA1919273	[1]	8.72	31.8
JM-09-86	thxn.JM86	*H. timareta thelxinoe*	Peru	m	-6.4528	-76.2987	SAMEA1919263	[1]	11.06	40.33
MJ12-3221	thxn.MJ12-3221	*H. timareta thelxinoe*	Peru	m	-5.6546	-77.6938	SAMEA104585110	[81]	6.74	24.58
MJ12-3233	thxn.MJ12-3233	*H. timareta thelxinoe*	Peru	m	-6.4519	-76.2985	SAMEA104585111	[81]	6.80	24.79
MJ12-3308	thxn.MJ12-3308	*H. timareta thelxinoe*	Peru	m	-5.6546	-77.6938	SAMEA104585112	[81]	6.57	23.95
MJ11-3339	txn.MJ11-3339	*H. timareta*	Peru	m	-5.6546	-77.6938	SAMEA104585113	[81]	7.25	26.43

		thelxinoe								
MJ11-3340	txn.MJ11-3340	*H. timareta thelxinoe*	Peru	m	-5.6546	-77.6938	SAMEA104585114	[81]	6.34	23.12
MJ11-3460	txn.MJ12-3460	*H. timareta thelxinoe*	Peru	m	-5.6546	-77.6938	SAMEA104585115	[81]	10.55	38.46
CS002395	flo.CS12	*H. timareta florencia*	Colombia	m	1.7097	-75.6976	SAMEA104585100	[81]	7.77	28.35
CS002402	flo.CS13	*H. timareta florencia*	Colombia	m	1.7097	-75.6976	SAMEA104585101	[81]	7.41	27.03
CS002403	flo.CS14	*H. timareta florencia*	Colombia	m	1.7097	-75.6976	SAMEA104585102	[81]	8.34	30.4
CS002406	flo.CS15	*H. timareta florencia*	Colombia	m	1.7097	-75.6976	SAMEA104585103	[81]	8.30	30.27
CS002337	flo.CS2337	*H. timareta florencia*	Colombia	m	1.7108	-75.7089	SAMEA104585104	[81]	11.99	43.74
CS002338	flo.CS2338	*H. timareta florencia*	Colombia	m	1.7108	-75.7089	SAMEA104585105	[81]	6.48	23.63
CS002341	flo.CS2341	*H. timareta florencia*	Colombia	m	1.8136	-75.6686	SAMEA104585106	[81]	7.90	28.81
CS002350	flo.CS2350	*H. timareta florencia*	Colombia	m	1.7108	-75.7089	SAMEA104585107	[81]	6.73	24.53
CS002358	flo.CS2358	*H. timareta florencia*	Colombia	m	1.7108	-75.7089	SAMEA104585108	[81]	7.16	26.12
CS002359	flo.CS2359	*H. timareta florencia*	Colombia	m	1.7108	-75.7089	SAMEA104585109	[81]	6.33	23.07
CAM001841	ros.CAM1841	*H. melpomene rosina*	Panama	m	9.0760	-79.6590	SAMEA104585083	[81]	7.53	27.47
CAM001880	ros.CAM1880	*H. melpomene rosina*	Panama	m	9.0760	-79.6590	SAMEA104585084	[81]	8.39	30.59
CAM002045	ros.CAM2045	*H. melpomene rosina*	Panama	m	9.1103	-79.6907	SAMEA104585085	[81]	6.24	22.76
CAM002059	ros.CAM2059	*H. melpomene rosina*	Panama	m	9.1103	-79.6907	SAMEA104585086	[81]	7.05	25.7
CAM002519	ros.CAM2519	*H. melpomene rosina*	Panama	m	9.0109	-79.5477	SAMEA104585087	[81]	7.68	28
CAM002552	ros.CAM2552	*H. melpomene rosina*	Panama	m	9.0109	-79.5477	SAMEA104585088	[81]	6.53	23.83
CAM002071	ros.CJ2071	*H. melpomene rosina*	Panama	m	9.1206	-79.6969	SAMEA1919257	[1]	10.08	36.77
CAM000531	ros.CJ531	*H. melpomene rosina*	Panama	m	9.1206	-79.6969	SAMEA1919271	[1]	7.36	26.83
CAM000533	ros.CJ533	*H. melpomene rosina*	Panama	m	9.1206	-79.6969	SAMEA1919260	[1]	7.31	26.68
CAM000546	ros.CJ546	*H. melpomene rosina*	Panama	m	9.1206	-79.6969	SAMEA1919279	[1]	7.25	26.43
CS000710	vul.CS10	*H. melpomene vulcanus*	Colombia	m	3.9000	-76.6325	SAMEA3723391	[49]	9.76	35.59
CS003603	vul.CS3603	*H. melpomene vulcanus*	Colombia	m	3.5175	-76.7572	SAMEA104585091	[81]	8.30	30.25
CS003605	vul.CS3605	*H. melpomene vulcanus*	Colombia	m	3.5175	-76.7572	SAMEA104585092	[81]	7.55	27.55
CS003606	vul.CS3606	*H. melpomene vulcanus*	Colombia	m	3.5175	-76.7572	SAMEA104585093	[81]	7.31	26.66
CS003612	vul.CS3612	*H. melpomene vulcanus*	Colombia	m	3.5175	-76.7572	SAMEA104585094	[81]	9.07	33.06
CS003614	vul.CS3614	*H. melpomene vulcanus*	Colombia	m	3.5175	-76.7572	SAMEA104585095	[81]	7.49	27.33
CS003615	vul.CS3615	*H. melpomene vulcanus*	Colombia	m	3.5175	-76.7572	SAMEA104585096	[81]	6.44	23.5

CS003617	vul.CS3617	*H. melpomene vulcanus*	Colombia	m	3.5175	-76.7572	SAMEA104585097	[81]	6.92	25.22
CS003618	vul.CS3618	*H. melpomene vulcanus*	Colombia	m	3.5175	-76.7572	SAMEA104585098	[81]	6.92	25.24
CS003621	vul.CS3621	*H. melpomene vulcanus*	Colombia	m	3.5175	-76.7572	SAMEA104585099	[81]	6.25	22.8
CS001002	mal.CS1002	*H. melpomene malleti*	Colombia	m	1.8033	-75.6553	SAMEA104585067	[81]	6.27	22.85
CS001011	mal.CS1011	*H. melpomene malleti*	Colombia	m	1.8033	-75.6553	SAMEA104585068	[81]	7.76	28.29
CS001815	mal.CS1815	*H. melpomene malleti*	Colombia	m	1.8033	-75.6553	SAMEA104585069	[81]	5.82	21.21
CS002311	mal.CS21	*H. melpomene malleti*	Colombia	m	1.8136	-75.6686	SAMEA3723397	[49]	9.75	35.56
CS001286	mal.CS22	*H. melpomene malleti*	Colombia	m	1.6097	-75.6669	SAMEA3723398	[49]	8.78	32.01
CS001321	mal.CS24	*H. melpomene malleti*	Colombia	m	1.7506	-75.6319	SAMEA3723399	[49]	7.47	27.25
CS000586	mal.CS586	*H. melpomene malleti*	Colombia	m	1.8033	-75.6553	SAMEA104585071	[81]	6.55	23.87
CS000594	mal.CS594	*H. melpomene malleti*	Colombia	f	1.8033	-75.6553	SAMEA104585072	[81]	9.36	34.12
CS000604	mal.CS604	*H. melpomene malleti*	Colombia	m	1.8033	-75.6553	SAMEA104585073	[81]	6.99	25.48
CS000615	mal.CS615	*H. melpomene malleti*	Colombia	m	1.8033	-75.6553	SAMEA104585074	[81]	5.93	21.62
JM-11-160	ama.JM160	*H. melpomene amaryllis*	Peru	f	-5.6756	-77.6747	SAMEA1919261	[1]	12.08	44.05
JM-09-216	ama.JM216	*H. melpomene amaryllis*	Peru	m	-6.4685	-76.3533	SAMEA1919261	[1]	8.89	32.41
JM-11-293	ama.JM293	*H. melpomene amaryllis*	Peru	f	-6.4703	-76.3473	SAMEA1919277	[1]	14.67	53.5
JM-11-48	ama.JM48	*H. melpomene amaryllis*	Peru	m	-6.0960	-76.9774	SAMEA1919269	[1]	15.30	55.81
MJ11-3188	ama.MJ11-3188	*H. melpomene amaryllis*	Peru	m	-5.6728	-77.7195	SAMEA104585061	[81]	6.87	25.05
MJ11-3189	ama.MJ11-3189	*H. melpomene amaryllis*	Peru	m	-5.6728	-77.7195	SAMEA104585062	[81]	7.66	27.95
MJ11-3202	ama.MJ11-3202	*H. melpomene amaryllis*	Peru	m	-5.6745	-77.6711	SAMEA104585063	[81]	6.19	22.58
MJ12-3217	ama.MJ12-3217	*H. melpomene amaryllis*	Peru	m	-6.4547	-76.2994	SAMEA104585064	[81]	7.04	25.67
MJ12-3258	ama.MJ12-3258	*H. melpomene amaryllis*	Peru	m	-6.4530	-76.2876	SAMEA104585065	[81]	6.79	24.77
MJ12-3301	ama.MJ12-3301	*H. melpomene amaryllis*	Peru	m	-6.4528	-76.2862	SAMEA104585066	[81]	6.39	23.29
CAM001349	melG.CAM1349	*H. melpomene melpomene*	French Guiana	f	2.5222	-51.1934	SAMEA104585075	[81]	6.44	23.49
CAM001422	melG.CAM1422	*H. melpomene melpomene*	French Guiana	m	2.5222	-51.1934	SAMEA104585076	[81]	10.00	36.47
CAM002035	melG.CAM2035	*H. melpomene melpomene*	French Guiana	m	2.5222	-51.1934	SAMEA104585077	[81]	7.19	26.24
CAM008171	melG.CAM8171	*H. melpomene melpomene*	French Guiana	f	2.5222	-51.1934	SAMEA104585078	[81]	7.01	25.57
CAM008216	melG.CAM8216	*H. melpomene melpomene*	French Guiana	m	2.5222	-51.1934	SAMEA104585080	[81]	7.84	28.57
CAM008218	melG.CAM8218	*H. melpomene melpomene*	French Guiana	m	2.5222	-51.1934	SAMEA104585081	[81]	6.24	22.74
CAM013435	melG.CJ13435	*H. melpomene melpomene*	French Guiana	m	2.5222	-51.1934	SAMEA1919276	[1]	9.82	35.81

CAM009315	melG.CJ9315	*H. melpomene melpomene*	French Guiana	m	2.5222	-51.1934	SAMEA1919270	[1]	6.65	24.26
CAM009316	melG.CJ9316	*H. melpomene melpomene*	French Guiana	m	2.5222	-51.1934	SAMEA1919252	[1]	6.35	23.15
CAM009317	melG.CJ9317	*H. melpomene melpomene*	French Guiana	m	2.5222	-51.1934	SAMEA1919267	[1]	9.64	35.14
MJ09-4125	MJ09.4125	*H. numata numata*	French Guiana		4.0833	-52.6753	SAMEA3888884	[65]	6.31	22
MJ09-4184	MJ09.4184	*H. numata silvana*	French Guiana		4.0834	-52.6753	SAMEA3888889	[65]	7.71	27.11
