# Supplementary material for: Recombination rate variation shapes barriers to introgression across butterfly genomes
Source: PLoS Biol. 2019 Feb 7;17(2):e2006288. doi: 10.1371/journal.pbio.2006288 (PMC6366726; doi:10.1371/journal.pbio.2006288)
Supplement: S2 Table — (PDF) [file pbio.2006288.s015.pdf]

S2 Table. Correlations between admixture proportion (f_d) and recombination rate

A. Population recombination rate (ρ)

	population set	all windows Spearman's rho	all windows p-value	thinned windows Spearman's rho	thinned windows p-value
whole genome	1 (*cyd* / *mel-W*)	0.346	**1.13E-73**	0.351	**4.09E-39**
	4 (*tim* / *mel-E*)	0.484	**2.43E-152**	0.494	**8.17E-81**
intermediate rec.	1 (*cyd* / *mel-W*)	0.188	**3.77E-14**	0.158	**4.63E-06**
	4 (*tim* / *mel-E*)	0.279	**1.06E-29**	0.298	**1.11E-17**
fused chromosomes	1 (*cyd* / *mel-W*)	0.288	**2.05E-32**	0.299	**2.38E-18**
	4 (*tim* / *mel-E*)	0.437	**5.02E-76**	0.439	**1.86E-39**
unfused chromosomes	1 (*cyd* / *mel-W*)	0.303	**1.79E-22**	0.322	**1.16E-13**
	4 (*tim* / *mel-E*)	0.431	**8.00E-46**	0.450	**3.33E-26**

B. Crossover recombination rate

	population set	all windows Spearman's rho	all windows p-value	thinned windows Spearman's rho	thinned windows p-value
whole genome	1 (*cyd* / *mel-W*)	0.280	**4.17E-48**	0.261	**5.54E-22**
	4 (*tim* / *mel-E*)	0.349	**2.69E-75**	0.358	**9.58E-41**
intermediate rec.	1 (*cyd* / *mel-W*)	0.129	**1.52E-07**	0.078	**1.40E-02**
	4 (*tim* / *mel-E*)	0.146	**3.14E-09**	0.138	**5.78E-05**
fused chromosomes	1 (*cyd* / *mel-W*)	0.189	**1.14E-14**	0.190	**2.94E-08**
	4 (*tim* / *melE*)	0.260	**1.58E-26**	0.259	**3.96E-14**
unfused chromosomes	1 (*cyd* / *mel-W*)	0.197	**2.67E-10**	0.175	**4.88E-05**
	4 (*tim* / *mel-E*)	0.202	**9.49E-11**	0.211	**1.23E-06**
